# Dynamics and Embedded Internet of Things Input Shaping Control for Overhead Cranes Transporting Multibody Payloads

**DOI:** 10.3390/s18061817

**Published:** 2018-06-04

**Authors:** Gerardo Peláez, Joshua Vaugan, Pablo Izquierdo, Higinio Rubio, Juan Carlos García-Prada

**Affiliations:** 1 Department of Mechanical Engineering, Universidade de Vigo, 36310 Pontevedra, Spain; pabloizquierdob@uvigo.es; 2 Department of Mechanical Engineering, University of Louisiana at Lafayette, Lafayette, LA 70504, USA; joshua.vaughan@louisiana.edu; 3 Department of Mechanical Engineering, Universidad Carlos III de Madrid, 28903 Madrid, Spain; hrubio@ing.uc3m.es (H.R.); jcgprada@ing.uc3m.es (J.C.G.-P.)

**Keywords:** Input Shaping, multibody, multimode, Embedded-IoT, feedforward real-time control, Functional Mock-Up Interface (FMI), video processing, Wireless Data Transmission 802.14.5 radios

## Abstract

Input shaping is an Optimal Control feedforward strategy whose ability to define how and when a flexible dynamical system defined by Ordinary Differential Equations (ODEs) and computer controlled would move into its operative space, without command induced unwanted dynamics, has been exhaustively demonstrated. This work examines the issue of Embedded Internet of Things (IoT) Input Shaping with regard to real time control of multibody oscillatory systems whose dynamics are better described by differential algebraic equations (DAEs). An overhead crane hanging a double link multibody payload has been appointed as a benchmark case; it is a multibody, multimode system. This might be worst scenario to implement Input Shaping. The reasons can be found in the wide array of constraints that arise. Firstly, the reliability of the multibody model was tested on a Functional Mock-Up Interface (FMI) with the two link payload suspended from the trolley by comparing the experimental video tapping signals in time domain faced with the signals extracted from the multibody model. The FFTs of the simulated and the experimental signal contain the same frequency harmonics only with somewhat different power due to the real world light damping in the joints. The application of this approach may be extended to other cases i.e., the usefulness of mobile hydraulic cranes is limited because the payload is supported by an overhead cable under tension that allows oscillation to occur during crane motion. If the payload size is not negligible small when compared with the cable length may introduce an additional oscillatory mode that creates a multibody double pendulum. To give the insight into the double pendulum dynamics by Lagrangian methods two slender rods as payloads are analyzed dealing with the overhead crane and a composite revolute-revolute joint is proposed to model the cable of the hydraulic crane, both assumptions facilitates an affordable analysis. This allows developing a general study of this type of multibody payloads dynamics including its normal modes, modes ratios plus ranges of frequencies expected. Input Shapers were calculated for those multimodes of vibration by convolving Specified Insensitivity (SI) shapers for each mode plus a novel Direct SI-SI shaper well suited to reduce the computational requirements, i.e., the number of the shaper taps, to carry out the convolution sum in real time by the IoT device based on a single microcontroller working as the command generator. Several comparisons are presented for the shaped and unshaped responses using both the multibody model, the experimental FMI set-up and finally a real world hydraulic crane under slewing motion commanded by an analog Joystick connected by two RF modules 802.15.4 to the IoT device that carry out the convolution sum in real time. Input Shaping improves the performances for all the cases.

## 1. Introduction

Multibody dynamics analysis is a generalized mechanical engineering method that does not make any assumptions based on intuition. Therefore, it is the objective of a multibody strategy to introduce computational techniques that can be applied methodically to dynamic systems composed of bodies undergoing spatial or planar motions in order to accurately carry out their dynamic response, regardless of their complexity [1,2,3].

**Figure 1:**
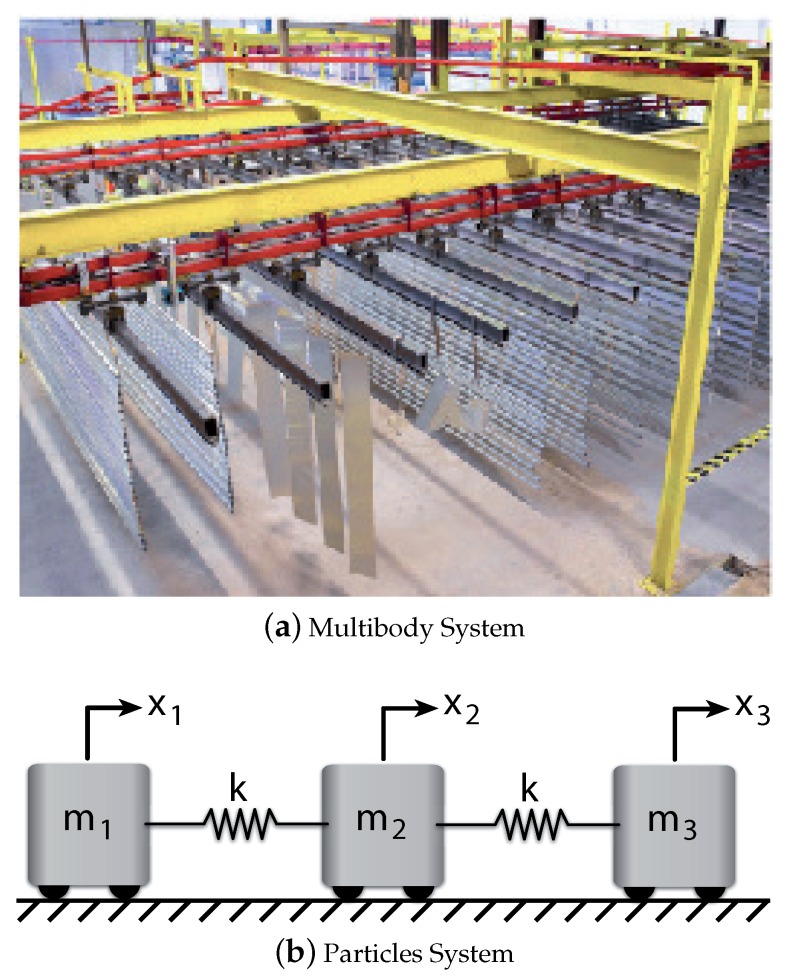
(**a**) Bodies that interact with each other through joints. (**b**) Particles that interact with each other throught forces.

Figure 1 depicts two systems composed of several bodies. The first system consisting of three moving bodies, the trolley plus a payload formed by two rigid links, might constitute a multibody model of an overhead crane. The bodies of this system interact with each other through translational-plus-revolute joints. The kinematic constraint equations corresponding to the joints set up a dependence between the bodies coordinates, so the overhead crane mechanism can be referred to as a three Degree Of Freedom multibody system. The second system is also a (3-DOF), but the bodies do not reach each other through joints but forces. In other words, the spring-mass system can not be considered strictly as a multibody system, and it is customary to model this system considering the masses as particles Figure 1b. Both systems exhibit an underdamped response, so mechanical vibration occurs when moving without taking into account such flexibility. The corresponding oscillatory responses can be determined using a particle oriented model regarding the three masses and springs system or a well suited multibody model for the overhead crane case.

**Figure 2:**
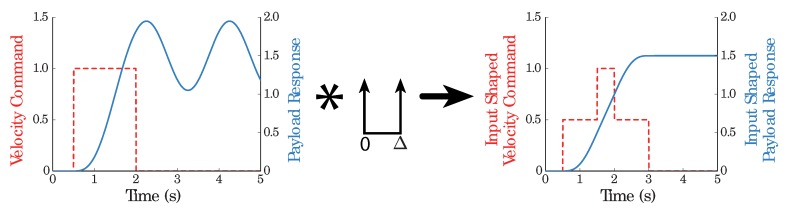
Step response, Posicast shaped command and resulting output.

The overhead cranes referred to above feed components to warehouses and manufacturing cells in industry. Such operations usually involve moving distributed, multibody payloads. Mitigating the transient sway helps avoid the collisions between such payloads and surroundings. Cancelling the residual vibration at the end of the motion facilitates the operations of loading and unloading. Obviously, overcoming such vibration benefits the performance. Overhead cranes are only a small group of the wide array of crane systems. Subsequently, it is not surprising that a quite valuable amount of research and development has faced the flexibility problem, thinking about improving human operation and performances of crane systems that exhibit oscillatory dynamics. Attenuating vibration induced during the maneuvering of oscillatory mechanical systems by designing the command reference signal profile is one of the most appealing solutions adopted [4,5,6]. The generalized term formally accepted for this strategy is command shaping. The method requires only estimates of the natural frequencies and damping ratio parameters. The apparent simplicity regarding the identification of these parameters, dealing with linear systems whose dynamics is described by ordinary differential equations (ODEs), allows it to be used in a straightforward manner as shown in Figure 2. In this figure a step input is convolved with a two impulse sequence, and resulting convolution sum causes no system vibration. In fact, the two mass and spring particle oriented model has become the command shaping benchmark system. The intuitive Newtonian laws application is the more customary option to obtain the ordinary differential equations describing the dynamics for such type of linear systems in the command shaping research [7,8,9,10,11,12,13,14].

Nonetheless, there is a wide array of mechanical systems, composed by bodies in contact by kinematic joints, that exhibit oscillatory responses and represent good candidates to apply Input Shaping. For these systems a multibody model is more accurate and performs better than a simple particle-oriented model. The contribution of this work is in the application of Input Shaping to this group of multibody systems, whose dynamics are described by Differential Algebraic equations (DAEs) rather than (ODEs) [3]. The overhead cranes hanging distributed multibody payloads are only one case.

There is a reduced group of works relative to the application of Input Shaping dealing with actual multibody systems whose dynamics is described by DAEs models [15], where the identification of the natural frequencies and damping ratios is not as straightforward to obtain as in the case of linear systems described by ODEs models [16,17,18]. In addition, the difficulties increase if only an Internet of Things (IoT) device—microcontroller-based—is available as command generator instead of a powerful computer. If this is the case, one has to resort to Direct Specified Insensitive Shapers filters with the shorter number of taps keeping unchanged the robustness for the frequencies ranges to be mitigated. This is the challenge that this work faces with. However, this is the real world case for a wide array of cranes.

## 2. Contributions

In words, the contribution of this work is to make embedded IoT input shaping real-time control well suited for multibody-multimode systems whose dynamics is described by DAEs according to a reliable set of solutions.

- First, the kinematic joints constraint equations of the multibody system model are written in compact form using generalized coordinates ϕ[c]=0. Thus, deriving, the velocity constraints are obtained arising the corresponding Jacobian matrix of the multibody model DAEs $\dot{\phi }=D\dot{c}=0$.
- The reliability of the multibody model is validated facing the simulated responses with the experimental signal of the real-world responses, extracted by video-tapping the functional mock up interface (FMI) motion.
- The FFTs of the experimental and the simulated signals contain the same frequency harmonics only with somewhat different power due to some real world light damping in the joints. Nonetheless the high degree correspondence between the signal spectrums guarantees the reliable multibody system flexible modes of vibration identification.
- To give some insight into the multibody double pendulum dynamics by Lagrangian methods a somewhat simplified, using a composite revolute-revolute joint, two link benchmark multibody model, is proposed. This allows to develop a general study of this type of multibody payloads dynamics including its normal modes, modes ratios plus ranges of frequencies expected.
- A robust Direct Specified Insenstivity (DSI) shaper is synthesized for the frequency ranges to be cancelled with help of the pair GAMS-CONOPT a proven choice for highly nonlinear problems. The DSI shaper is robust plus optimal, regarding the number of the filter taps, to be implemented in an embedded microcontroller working as the system command generator that carry out the convolution sum in real-time.
- The DSI shaper robustness performance is tested by simulation using the multibody model plus facing the DSI burned microcontroller working as the command generator of the real world FMI set up and the hydraulic crane.

The next two sections of this work brief review the synthesis of robust input shapers in addition describes how the multibody model was formed. Section 5 deals with the validation, by facing the numerical simulated responses with the experimental response of a Functional Mock-Up Interface of an overhead crane handling two links as payload. Secondly the two-modes of vibration are analytical studied with the help of a proposed benchmark model applying Lagrangian methods. The Section 6 documents and illustrates the tailored features and advantages of DSI embedded IoT command shaping to improve the dynamics of overhead cranes transporting multibody payloads. The Section 7 compares simulation and experimental results coming from the FMI plus a real world hydraulic crane. Finally some conclusions are given in the last section.

## 3. Conceptualization: Robust Input Shaping

**Figure 3:**
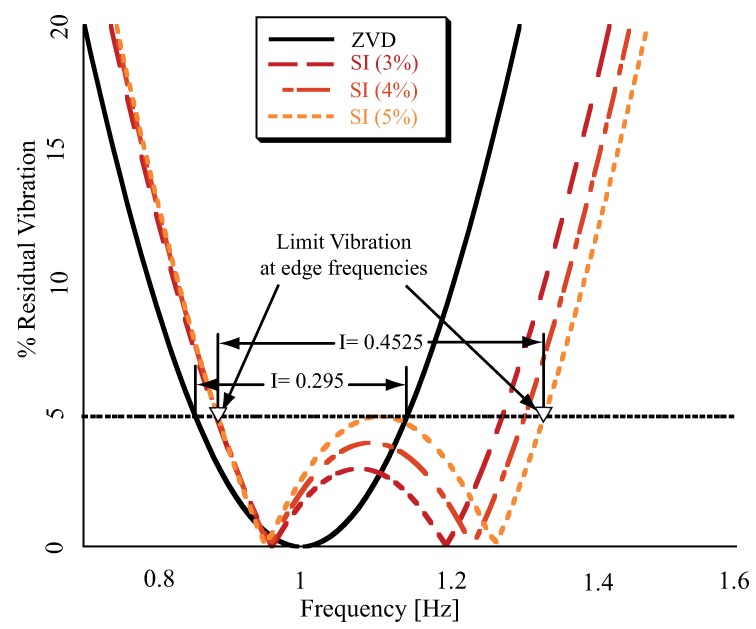
Robust input shapers sensitivity curves as a function of the vibration limit.

The Figure 3 shows several sensitivity curves, where the amplitude of the residual vibration is plotted as a function of the actual frequency. The vertical axis is the percentage of vibration given by Equation (1), while the horizontal axis is the frequency range considered, corresponding to the system natural frequency. Notice that the residual vibration of the Zero Vibration and Derivative (ZVD) shaper, shown as a black line, increases rapidly as the actual frequency deviates from the central frequency of around 1 Hz. The ZVD shaper, can be synthesized from Equation (1) applying the constraints: $V_{tol}=0$ plus $\frac{dV_{tol}}{d\omega }=0$.

The shaper’s robustness can be measured quantitatively by measuring the width of the curve at some low level of tolerable vibration $V_{tol}$. This non-dimensional parameter is called the shaper’s frequency insensitivity I. $V_{tol}$ can be written as:

(1) $$V_{tol}⩾e^{-\zeta \omega T_{n}}\cdot \sqrt{(C{\left(\omega ,\zeta \right)}^{2}+S{\left(\omega ,\zeta \right)}^{2}}$$

where

(2) $$C\left(\omega ,\zeta \right)=\sum e^{\zeta \omega T_{i}}\cdot A_{i}\cdot \cos\left(\omega \sqrt{1-\delta^{2}}T_{i}\right)$$

(3) $$S\left(\omega ,\zeta \right)=\sum e^{\zeta \omega T_{i}}\cdot A_{i}\cdot \sin\left(\omega \sqrt{1-\delta^{2}}T_{i}\right)$$

In the Input Shaping research [16,19,20], this important equation can be found repeatedly. By using Equation (1) as a constraint equation, it is possible to set the residual vibration to a desired percentage of the pre-existing vibration, that is, the vibration that occurs without filtering with Input Shaping. As demonstrated in Figure 3, increased and selective robustness can be achieving if a Specified Insensitive (SI) shaper is designed to mitigate vibration at specified frequency ranges. The most straightforward method for generating a shaper with specified insensitivity to frequency uncertainty is the technique of frequency sampling [19]. This method implies repeated use of the also called residual energy Equation (1) at each one of a frequencies set, spaced at a regular sampling period, contained in the given frequency band. In each case, $V_{tol}\left(\omega ,\zeta \right)$ is set less or equal to the tolerable level of residual energy. If the limit $V_{tol}$ is relaxed, allowing some increase, then the robustness of the Shaper/Filter also increases, keeping unchanged the set of frequencies where such constrain is applied as demonstrates Figure 3.

An alternative strategy to generate SI Filters by frequency sampling, entails parameterizing the sensitivity curve plus the filter coefficients and solving for the optimal parameters via a gradient-based constraint optimization algorithm. If this is the case, the filter can be parameterized in terms of delay times and gains. For instance, the SI-shaper, can be expressed as:

(4) $$IS=A_{1}\delta \left(t\right)+A_{2}\delta \left(t-T_{s2}\right)+A_{3}\delta \left(t-T_{s3}\right)+A_{4}\delta \left(t-T_{s4}\right)$$

where $A_{1}$, $A_{2}$, $A_{3}$ and $A_{4}$ are the amplitudes of the Input Shaper taps, while $T_{s2}$, $T_{s3}$ and $T_{s4}$ are the times where the impulses or filter taps are applied. Here, it is assumed that the first impulse is applied at time zero, in order to minimize the delay induced by the filter. In this sense, the contribution of Tarunraj Singh research must be referenced [21]. Singh proposed Time Delay Filters (TDF), which are insensitive to different uncertainties, the stiffness especially, and posed them in the framework of a minimax optimization problem [22,23]. His work converges with Singhose previous works plus results [24] both remarkable. In addition, the other aforementioned parameters are the unknown frequency $\omega_{h1}$ at which the peak of the sensitivity curve occurs plus the touch frequencies $\omega_{z1}$, $\omega_{z2}$ where the SI-filter sensitivity curve must be forced to be zero between the frequency $\omega_{h1}$ where the hump of the sensitivity curve takes place, i.e., the slope equals zero. At this point all the parameters to be determined have been enumerated. The resulting non-linear programming is:

objective

(5) $$minimize\to T_{sf}$$

subject to

(6) $$V_{tol}⩾e^{\left(-\zeta \omega_{e1}T_{sf}\right)}\sqrt{{\left(C\left(\omega_{e1},\zeta \right)\right)}^{2}+{\left(S\left(\omega_{e1},\zeta \right)\right)}^{2}}\cdots \omega_{e1}=\left(1-\frac{I}{2}\right)\omega_{m}$$

(7) $$V_{tol}⩾e^{\left(-\zeta \omega_{e2}T_{sf}\right)}\sqrt{{\left(C\left(\omega_{e2},\zeta \right)\right)}^{2}+{\left(S\left(\omega_{e2},\zeta \right)\right)}^{2}}\cdots \omega_{e2}=\left(1+\frac{I}{2}\right)\omega_{m}$$

(8) $$V_{tol}=e^{\left(-\zeta \omega_{h1}T_{sf}\right)}\sqrt{{\left(C\left(\omega_{h1},\zeta \right)\right)}^{2}+{\left(S\left(\omega_{h1},\zeta \right)\right)}^{2}}$$

(9) $$0=\frac{d}{d\omega }\left[e^{\left(-\zeta \omega_{h1}T_{sf}\right)}\sqrt{{\left(C\left(\omega_{h1},\zeta \right)\right)}^{2}+{\left(S\left(\omega_{h1},\zeta \right)\right)}^{2}}\right]$$

(10) $$0=e^{\left(-\zeta \omega_{z1}T_{sf}\right)}\sqrt{{\left(C\left(\omega_{z1},\zeta \right)\right)}^{2}+{\left(S\left(\omega_{z1},\zeta \right)\right)}^{2}}$$

(11) $$0=e^{\left(-\zeta \omega_{z2}T_{sf}\right)}\sqrt{{\left(C\left(\omega_{z2},\zeta \right)\right)}^{2}+{\left(S\left(\omega_{z2},\zeta \right)\right)}^{2}}$$

where $\omega_{z1}$, $\omega_{z2}$ are the unknown frequencies that interweave the edge and hump frequency $\omega_{h1}$, delivering another set of constraints

(12) $$\left(1-\frac{I}{2}\right)\omega_{m}<\omega_{z1}<\omega_{h1}<\omega_{z2}<\left(1+\frac{I}{2}\right)\omega_{m}$$

Finally, the amplitudes must sum to one, in order to the shaped command will reach the same final set point as the unshaped command.

(13) $$1=\Sigma_{i}A_{i}$$

Interest in Input Shaping was increased by Singhose’s development of a very robust insensitive to modeling errors input shaper, named as Extra Insensitive (EI), [24]. Later on, procedures for precisely specifying the degree of robustness were presented. Characteristics of the impulse sequence as a function of robustness and system damping were discussed by in [16]. Dealing with the aforementioned work, Singhose introduced a second more sophisticated procedure, that yields exact solutions. All these works assume that the frequency range where the benchmark second order system tend to vibrate can be easily estimated from the ordinary second order differential equation.

Dooroo Kim, Joshua Vaughan and William Singhose in [17] point to that the double particle pendulum payload dynamics introduce an additional oscillatory mode. As will be proved later some multibody systems pertain to the same array of multimode systems. If this is the case, these systems require increased robustness which is significantly different than that available with the standard input shapers i.e., ZV, ZVD, EI. In view of this, procedures to precisely set up the constraint equations that must be satisfied do not remain fixed, rather they vary with the desired level of insensitivity and frequencies ranges to be cancelled.

The Quantitative Feedback Input Shaping technique addresses nonlinear and time-varying systems even a trade-off to vibration induced by disturbance and noise can be quantifiable with this technique. Unfortunately, large and powerful computers are needed [25].

Advances in robustness dealing with actual trapezoidal profiles instead of theoretical pulses corresponding to Bang-Bang optimal time negative input shaping are remarkable as in the work of [26], however the control fall on proprietary closed hardware.

For a more detailed review dealing with the history of Input Shaping/Time Delay filtering theory, readers are referred to the work of John Y. Hung [27], where the basic principles of Posicast theory [28], some of its past history, and new trends and fields of application are presented. Also, readers might consult [29,30]. Recently Singhose, Kivila and Seering develop an interactive textbook dealing with command shaping methods to exploit the interactive functions of tablets [31].

## 4. Overhead Crane Multibody Model

**Figure 4:**
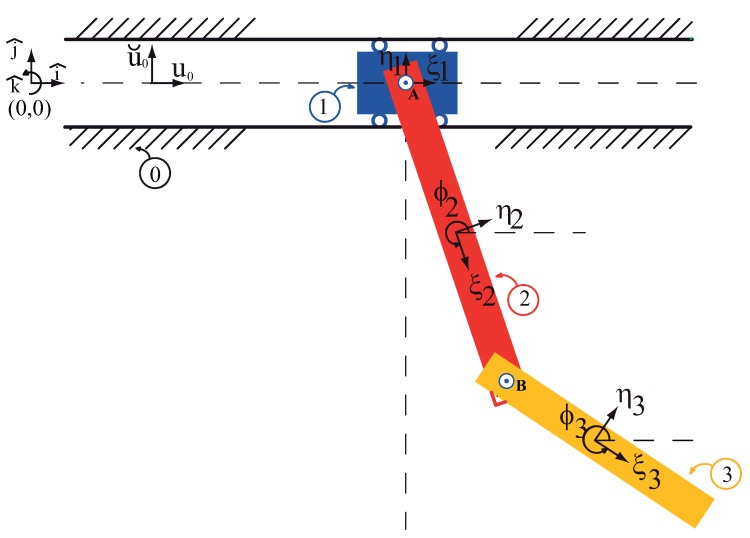
The overhead crane: the trolley plus the two-link payload, modeled as a multibody system.

**Figure 5:**
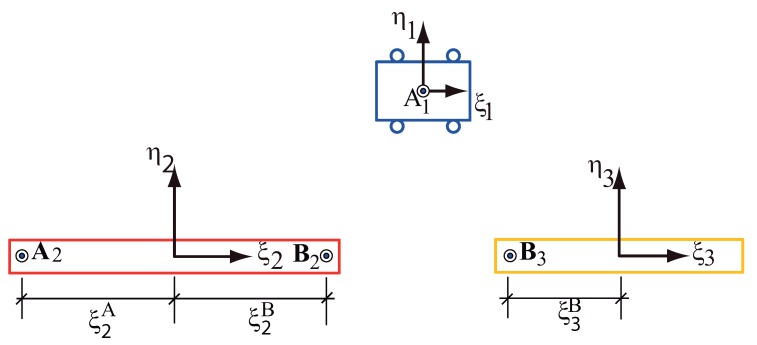
The multibody system viewed as an unconstrained system.

The multibody system of Figure 4 contains three moving bodies: the trolley numbered as ①, the first link numbered as ② and the second link of the payload numbered as ③. Also a non-moving x-y frame has been numbered as ⓪. Three body-fixed frames are also defined, assuming that the mass centres are known, the origins of the body frames are positioned at the mass centres. The pin joints are positioned with respect to their corresponding frame. As shows in Figure 5, there are:

1. A sliding joint between the body ①, the trolley, and the fixed frame.
2. A pin joint between the body ②, the first link, and the trolley, body ①.
3. A pin joint between the body ③, the second link, and the body ②, the first link.

The three moving bodies and ground, without considering the existence of the joints, form an unconstrained system shown in Figure 5. The *x*- and *y*-coordinates of the origin of each ξ−η frame describe the translational coordinates of that body. The angle between each ξ-axis and the *x*-axis describes the rotational coordinate of that body, $\phi_{i}$. Obviously, the presence of the kinematic joints, these variables no longer remain independent from one another, i.e., they are dependent coordinates.

From Figure 5, the constraint equation for the revolute joint at pin A ($A_{1}$ − $A_{2}$) is:

(14) $${}^{\left(r,2\right)}\Phi =r_{2}^{A}-r_{1}^{A}=r_{2}+s_{2}^{A}-r_{1}-s_{1}^{A}=0$$

where $s_{2}^{A}$ and $s_{1}^{A}$ are the vectors from the origin of the local frames to the point. These vectors can be written as a function of the local coordinates $\left(\xi_{2}^{A},\;\eta_{2}^{A}\right)$ of point A ($A_{1}$ − $A_{2}$) in the local axis of body 2, without loss of generality:

(15) $$s_{2}^{A}=\left\{\begin{array}{cc} cos\phi_{2} & -sin\phi_{2}\\ sin\phi_{2} & cos\phi_{2} \end{array}\right\}\cdot \left\{\begin{array}{c} \xi_{2}^{A}\\ \eta_{2}^{A} \end{array}\right\}$$

In view of this, the constraint expressed by Equation (14) can be expanded in the following form:

(16) $$\left\{\begin{array}{c} x_{2}\\ y_{2} \end{array}\right\}+\left\{\begin{array}{cc} cos\phi_{2} & -sin\phi_{2}\\ sin\phi_{2} & cos\phi_{2} \end{array}\right\}\cdot \left\{\begin{array}{c} \xi_{2}^{A}\\ \eta_{2}^{A} \end{array}\right\}-\left\{\begin{array}{c} x_{1}\\ y_{1} \end{array}\right\}=\left\{\begin{array}{c} 0\\ 0 \end{array}\right\}$$

The constraint equation for the revolute joint at pin B ($B_{2}$ − $B_{3}$) is written in compact form:

(17) $${}^{\left(r,2\right)}\Phi =r_{3}^{B}-r_{2}^{B}=0$$

Also, this constraint may be expanded by the same manner of the revolute joint A.

On the axis of the sliding joint, two points and one unit vector are needed to set up the constraint equation in a compact expression

(18) $${}^{\left(t,2\right)}\Phi =\left\{\begin{array}{c} \overset{˘}{u_{0}}\cdot \vec{d}\\ \phi_{1} \end{array}\right\}=0$$

where $\vec{u_{0}}=\left(1,0\right)$. Please note that this unit vector is referred to the global axis (*x*, *y*), thus its local coordinates obviously coincides with its global coordinates. Points O and A are used to construct the vector $\vec{d}=\vec{r_{1}}-\vec{r_{0}}$. Thus, $\vec{d}=\left\{x_{1}-0,y_{1}-0\right\}$. According to Equation (18) the dot product value between the vector *ŭ*, by the vector $\vec{d}$ is zero. As a result:

(19) $${}^{\left(t,2\right)}\Phi =\left\{\begin{array}{c} -u_{0y}d_{x}+u_{0x}d_{y}\\ \phi_{1} \end{array}\right\}=\left\{\begin{array}{c} -0\cdot x_{1}+1\cdot y_{1}\\ \phi_{1} \end{array}\right\}=\left\{\begin{array}{c} 0\\ 0 \end{array}\right\}$$

The sliding joint constraint implies a zero constant value for $y_{1}$ plus the angle between the local axis $\xi_{1}$ and the global axis *x*, i.e., the angle $\phi_{1}$ behaves zero during the whole motion. In addition to the kinematic constraints, the driver constraint would be considered because the motion of the trolley, body along the sliding axis, is defined by such constraint. Thus, its position depends on the value of a function of time shaped $f_{s}\left(t\right)$ or unshaped $f_{u}\left(t\right)$. This constraint can be expressed as:

(20) $${}^{\left(d-x_{1},1\right)}\Phi =\left\{x_{1}-f\left(t\right)\right\}=0$$

It is a rehonomous constraint. The equation of this constraint contains time as explicit variable. In view of this, in the following the appended constraint method is select to deal with it, apart from the escleronomous constraints introduced above [3] . The trolley plus the two links payload and the ground, without considering the existence of the referred joints and driver, form an unconstrained system, shown in Figure 5. The *x*- and *y*-coordinates of the origin of each ξ−η frame describes the translational coordinates of such body. The angle between each ξ-axis and the *x*-axis describes the rotational coordinates of the double link. These coordinates are arranged in three arrays as:

(21) $$c_{1}=\left\{\begin{array}{c} x_{1}\\ y_{1}\\ \phi_{1} \end{array}\right\},c_{2}=\left\{\begin{array}{c} x_{2}\\ y_{2}\\ \phi_{2} \end{array}\right\},c_{3}=\left\{\begin{array}{c} x_{3}\\ y_{3}\\ \phi_{3} \end{array}\right\}$$

These nine coordinates are variables; that is, if the bodies are allowed to move free in the plane, the coordinates find different values. In the presence of the kinematic joints described above, these variables are no longer independent from one another. The degrees of freedom of this mechanical system is three according to DOF = $n_{v}-n_{c}$ = 9 − 6 = 3, without considering the driver constraint. Six constraint equations correspond to the kinematic joints. The $n_{v}$ dependent coordinates defined as c are given by:

(22) $$c^{\prime }=\left\{\begin{array}{ccccccccc} x_{1} & y_{1} & \phi_{1} & x_{2} & y_{2} & \phi_{2} & x_{3} & y_{3} & \phi_{3} \end{array}\right\}$$

The position constraints introduced in Equations (14)–(19) can be expressed in general form as:

(23) Φ(c)=0

The velocity constrains are obtained from the time derivative of Equation (10)

(24) $$\dot{\Phi }=D\cdot \dot{c}=0$$

where the matrix *D* is the Jacobian of the constraint Equations (14)–(19). The Jacobian can be carried out step by step deriving such equations. The time derivative of the constraint equation of the revolute joint A in a compact form is given by:

(25) $${}^{\left(r,2\right)}\dot{\Phi }=\dot{r_{2}}+{\overset{˘}{s}}_{2}^{A}\cdot \dot{\phi_{2}}-\dot{r_{1}}$$

as a function of the local frames of reference this equation can be expanded to obtain the subjacobian of the revolute joint A:

(26) $${}^{\left(r,2\right)}\dot{\Phi }=\left\{\begin{array}{cccccc} -1 & 0 & 0 & 1 & 0 & -(\xi_{2}^{A}\cdot sin\phi_{2}+\eta_{2}^{A}\cdot cos\phi_{2})\\ 0 & -1 & 0 & 0 & 1 & \left(\xi_{2}^{A}\cdot cos\phi_{2}-\eta_{2}^{A}\cdot sin\phi_{2}\right) \end{array}\right\}\cdot \left\{\begin{array}{c} {\dot{x}}_{1}\\ {\dot{y}}_{1}\\ {\dot{\phi }}_{1}\\ {\dot{x}}_{2}\\ {\dot{y}}_{2}\\ {\dot{\phi }}_{2} \end{array}\right\}$$

The time derivative of the constraint equation corresponding to the revolute joint B in a compact form is:

(27) $${}^{\left(r,2\right)}\dot{\Phi }=\dot{r_{3}}+{\overset{˘}{s}}_{3}^{B}\cdot \dot{\phi_{3}}-\dot{r_{2}}-{\overset{˘}{s}}_{2}^{B}\cdot \dot{\phi_{2}}$$

Thus, as a function of the local frames of reference, this equation can be expanded to obtain the subjacobian corresponding to the revolute joint B:

(28) $$\dot{\Phi }=\left[\begin{array}{cccccc} -1 & 0 & \xi_{2}^{B}\sin\phi_{2}+\eta_{2}^{B}\cos\phi_{2} & 1 & 0 & -(\xi_{3}^{B}\sin\phi_{3}+\eta_{3}^{B}\cos\phi_{3})\\ 0 & -1 & -\xi_{2}^{B}\cos\phi_{2}+\eta_{2}^{B}\sin\phi_{2} & 0 & 1 & \xi_{3}^{B}\cos\phi_{3}-\eta_{3}^{B}\sin\phi_{3} \end{array}\right]\left[\begin{array}{c} {\dot{x}}_{2}\\ {\dot{y}}_{2}\\ {\dot{\phi }}_{2}\\ {\dot{x}}_{3}\\ {\dot{y}}_{3}\\ {\dot{\phi }}_{3} \end{array}\right]$$

Finally, the velocity constraints corresponding to the translational joint are:

(29) $${}^{\left(t,2\right)}\dot{\Phi }=\left\{\begin{array}{ccc} 0 & 1 & 0\\ 0 & 0 & 1 \end{array}\right\}\cdot \left\{\begin{array}{c} \dot{x_{1}}\\ \dot{y_{1}}\\ \dot{\phi_{1}} \end{array}\right\}$$

Equations (25)–(29) build the velocity constraints delivering the Jacobian matrix of the multibody system multiplied by the vector of the dependent variables:

(30) $$\begin{array}{c} \left\{\begin{array}{ccccccccc} 0 & 1 & 0 & 0 & 0 & 0 & 0 & 0 & 0\\ 0 & 0 & 1 & 0 & 0 & 0 & 0 & 0 & 0\\ -1 & 0 & 0 & 1 & 0 & -(\xi_{2}^{A}\cdot sin\phi_{2}+\eta_{2}^{A}\cdot cos\phi_{2}) & 0 & 0 & 0\\ 0 & -1 & 0 & 0 & 1 & \left(\xi_{2}^{A}\cdot cos\phi_{2}-\eta_{2}^{A}\cdot sin\phi_{2}\right) & 0 & 0 & 0\\ 0 & 0 & 0 & -1 & 0 & \left(\xi_{2}^{B}\cdot sin\phi_{2}+\eta_{2}^{B}\cdot cos\phi_{2}\right) & 1 & 0 & -(\xi_{3}^{B}\cdot sin\phi_{3}+\eta_{3}^{B}\cdot cos\phi_{3})\\ 0 & 0 & 0 & 0 & -1 & -(\xi_{2}^{B}\cdot cos\phi_{2}-\eta_{2}^{B}\cdot sin\phi_{2}) & 0 & 1 & \left(\xi_{3}^{B}\cdot cos\phi_{3}-\eta_{3}^{B}\cdot sin\phi_{3}\right) \end{array}\right\}\cdot \left\{\dot{c}\right\}=\left\{0\right\} \end{array}$$

This equation can be written in a more compact form as:

(31) $$\dot{\Phi }=D\cdot \dot{c}=0$$

deriving again, the acceleration constraint becomes:

(32) $$\ddot{\Phi }=D\cdot \ddot{c}+\dot{D}\cdot \dot{c}=D\cdot \ddot{c}-\gamma =0$$

The equations of motion for the unconstrained system of bodies shown in Figure 5 can be expressed as:

(33) $$M\ddot{c}=h$$

The equations of motion for the constrained overhead system depicted in Figure 4 must include the contact forces between the bodies. These contact forces can be modeled in a straightforward manner, with the help of the Jacobian plus the Lagrange multipliers, as follows:

(34) $$M\ddot{c}=h+D^{\prime }\cdot \lambda$$

These equations represent the dynamics of the set of constrained bodies formulated in body coordinates. If the unknowns are the accelerations, $\ddot{c}$, and Lagrange multipliers, λ, these equations are treated as linear algebraic equations. However, when the unknowns are the coordinates and velocities, the same equations must be treated as second-order differential-algebraic equations (DAE).

Algebraic equations like (34) must be solved for the accelerations. However, there are more unknowns in these equations than the number of equations. To make the number of equations equal to the number of unknowns, it is appended the acceleration constraints of (32)–(34). Rearranged in matrix form:

(35) $$\left[\begin{array}{cc} M & -D^{\prime }\\ D & 0 \end{array}\right]\cdot \left\{\begin{array}{c} \ddot{c}\\ \lambda \end{array}\right\}=\left\{\begin{array}{c} h\\ \gamma \end{array}\right\}$$

These algebraic equations can be solved for the unknows $\ddot{c}$ and λ and can be considered as the equations of motion for any arbitrary multibody system.

## 5. Validation of the Model

**Figure 6:**
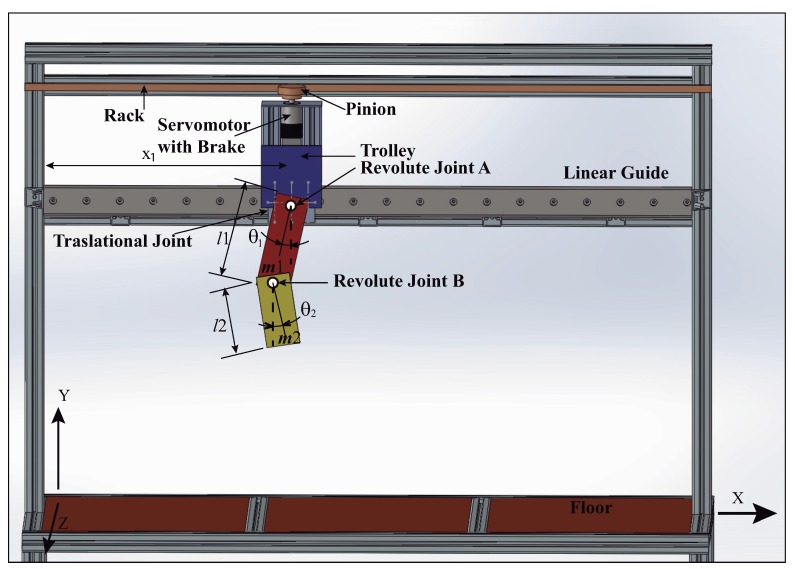
Sketch of the overhead crane Funtional Mock-Up Interface (FMI) with double pendulum multibody payload dynamics. Conditions: first link, length $l_{1}$ mass $m_{1}$, plus moment of inertia about the mass center $I_{zz}^{G1}$; second link, length $l_{2}$ mass $m_{2}$, plus moment of inertia about the mass center $I_{zz}^{G2}$.

The validation of this model can proceed in two ways. Facing the numerical simulation results with the experimental measurements extracted from a Functional Mock Up interface whose sketch is shown in Figure 6. Or comparing their numerical simulation results with the outcomes obtained by Lagrangian methods.

### 5.1. Validation of the Model Simulation Results Face to Face with Experimental FMI Outcomes

To give a more detailed description of the overhead crane with the double-pendulum multibody payload during motion, the position of the link-1 and link-2 within the Newtonian frame are written in the independent coordinates judiciously: $x_{1}$, $\theta_{1}$ and $\theta_{2}$ respectively as shows Figure 6 corresponding to the functional mock-up interface (FMI). The trolley moves lengthwise the linear guide, its position is described by $x_{1}$. As stated above the payload is composed by two rigid links plus two revolute joints. The link-1 of length $l_{1}$ hangs from the trolley via the revolute joint A, the second link-2 is connected to the link-1 via the revolute joint B. Both links are represented as rigid bodies having masses $m_{1}$, $m_{2}$ plus the corresponding inertial properties $I_{G1}$ and $I_{G2}$. The angles describing the deviations from vertical are shown in Figure 6. Notice that the angles $\theta_{1}$ and $\theta_{2}$ are defined by a vertical line in the *Y*-axis direction from the trolley to the floor.

The analytical equations of motion for this model are too complex to show in their entirety here, as they are not compact expressions. The most appealing option, as stated above consist in resort to the numerical integration of the multibody model DAEs, for some set of numerical values of the aforementioned parameters, they are depicted in Table 1.

There is a wide array of computational tools available in order to numerically integrate (35). MBS3D is an open-source general purpose program for the dynamic simulation of multibody systems. It is entirely programmed as plain text in Matlab and uses a very efficient and tested mathematical semi-recursive method due to García de Jalón [2]. Due to its popularity and easy of use, Matlab was chosen as the programming language for the routines that are developed in each chapter of Nikravesh textbook [3]. Those routines are available for engineers to form complete programs for a particular multibody system. Most of them have been used in this work to ensure simulations reliability by taking advantage of Nikravesh functions. If this is the case, the constraint violation stabilization using the gradient feedback or Baumgarte method is necessary, it is very effective and easy to implement in controlling of the geometric constrains [32]. Thus, the term:

(36) $$D\cdot \ddot{c}=-\dot{D}\cdot \dot{c}=\gamma$$

has been substituted by:

(37) $$D\cdot \ddot{c}=\gamma -2\alpha \dot{\Phi }-\beta^{2}\cdot \Phi$$

In general, for nonzero values of α and β, the numerical solution oscillates about the *correct* solution. Here, a value of α=β=20 has been adopted according to Paulo Flores works [33].

Finally, given the availability of the Matlab Simmechanics parser, the recent SolidWorks releases can export using ≪.xml≫ file format, the mates between the pieces plus the inertial properties of such pieces -treated as bodies-, to Matlab Simmechanics. Once this parser launch the ≪.xml≫ file, a simulink model is available to perform the dynamic simulation of the multibody system. In the present work this option has also been used to compare with the numerical integration of (35) by using Nikravesh Matlab functions plus procedures [3] .

**Figure 7:**
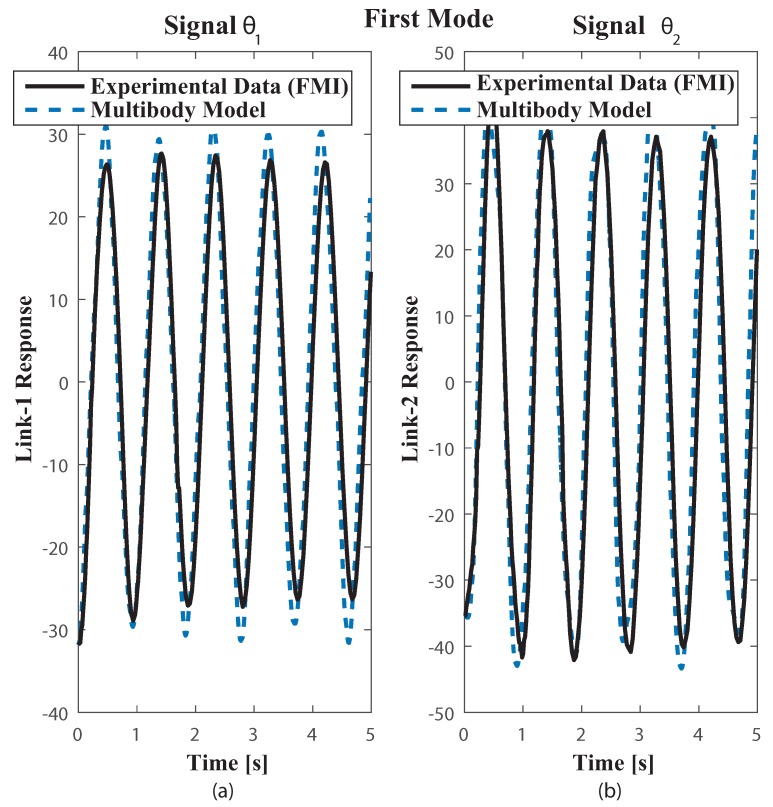
Plots of Experimental FMI and Multibody model free vibration motion responses closely to the first mode. Initial Conditions: first link deviation from vertical $\Theta_{1}$ = 31.84${}^{\circ }$, clockwise, second link deviation from vertical $\Theta_{2}$ = 26.39${}^{\circ }$, clockwise.

**Figure 8:**
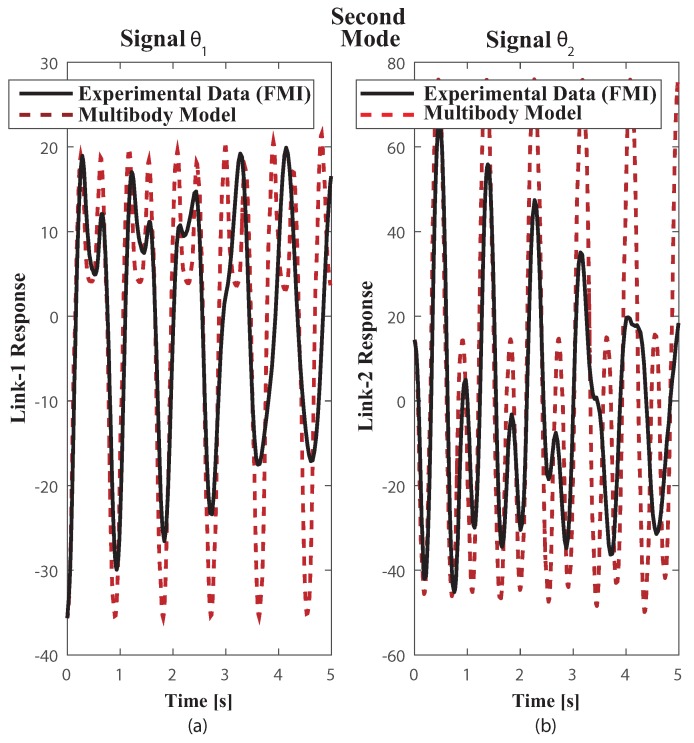
Plots of Experimental FMI and Multibody model free vibration motion responses closely to the second mode. Initial Conditions: first link from vertical $\Theta_{1}$ = 35.65${}^{\circ }$, clockwise, second link from vertical $\Theta_{1}$ = 14.42 ${}^{\circ }$ counter clockwise.

The multibody model was tested on the functional mock up interface (FMI) with the two link payload suspended from the trolley, using both measuring FMI real world system through a comparison of results obtained from the multibody model simulation as in [34]. The payload free vibration data was collected by video tapping the trajectory and measuring position of the mass centres plus revolute joints at each frame. The selected video recording speed was 60 fps. The payload response to initial conditions well suited to excite the first and second modes of vibration can be seen in the corresponding Figure 7 plus Figure 8. Dealing with the first mode both links oscillate closely to 30${}^{\circ }$ about the equilibrium position at a frequency of 1.1 Hz Figure 7. The second mode which is at 2.4 Hz shown in Figure 8 despite the actual experimental underdamped response when compared with the undamped multibody model response both behave closely. Nonetheless, as the number of vibration cycles increases the damping at joint B becomes relevant. This might cause some frequency mismatch, recall that $\omega_{d}=\omega_{n}\sqrt{1-\zeta^{2}}$, especially as the number of cycles increases.

**Figure 9:**
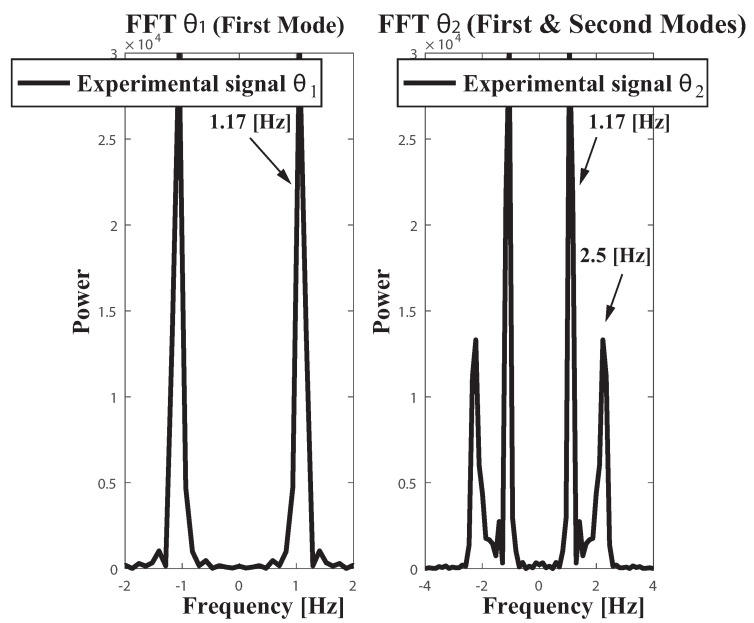
Fast Fourier Transform (FFT) of the experimental dynamic signals $\theta_{1}\left(t\right)$, $\theta_{2}\left(t\right)$. Conditions: sampling frequency 60 Hz.

**Figure 10:**
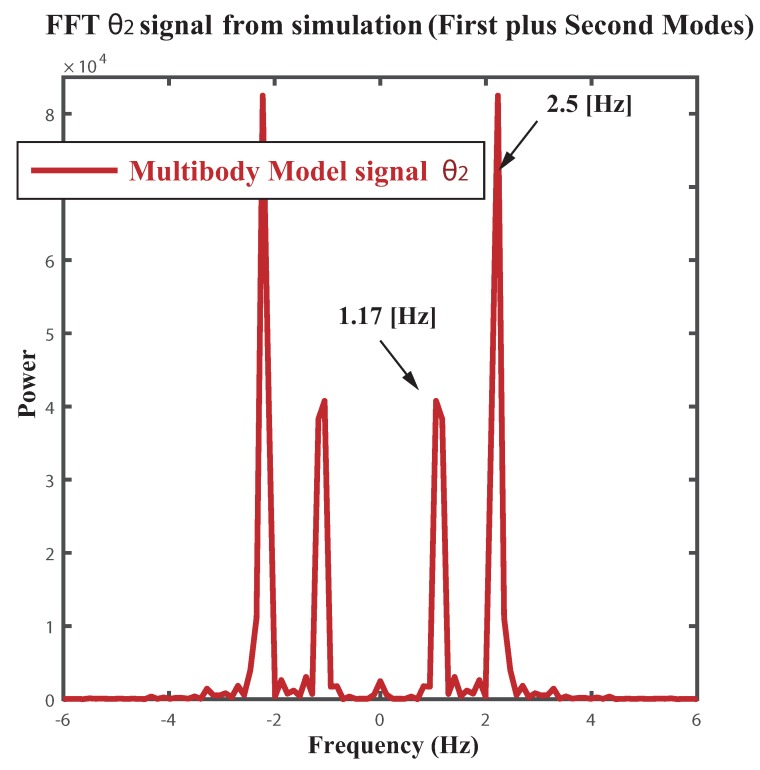
Fast Fourier Transform (FFT) of the simulated dynamic signal $\theta_{2}\left(t\right)$ . Conditions: Multibody model, sampling frequency 60 Hz.

The overall Figure 9 shows the Fast Fourier Transform (FFT) of the aforementioned experimental signals corresponding to video tapping both modes of vibration. To reinforce the notion about the reliability of the multibody model tunning for the experimental FMI, Figure 10 depicts the FFT of the dynamic signal $\theta_{2}$ corresponding to the second link deflection from vertical of the multibody model. Only differences dealing with the power of the first and second harmonics can be found between the experimental and the modeled FFT spectrum. Thus the reliability of the multibody model as a fair tool for the frequency analysis of a particular showcase has been proved.

Nonetheless the question about how is the general dynamics behavior of the multibody system remains still unanswered.

### 5.2. Lagrangian Analysis

**Figure 11:**
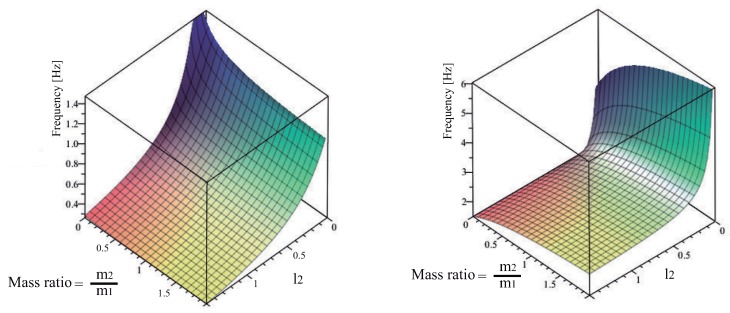
Variation of first and second mode frequencies when 0 < *l*${}_{2}$ < 1.5 and 0 < R < 2.

To give some insight into the multibody double pendulum dynamics by analytical methods the initial goal consist in develop the free vibration analysis considering negligible small damping in the joints of the multibody system. From the corresponding Euler-Lagrange equations

(38) $$\frac{d}{dt}\frac{\partial T}{\partial \dot{\theta_{i}}}-\frac{\partial (T-V)}{\partial \theta_{i}}+\frac{\partial F}{\partial \dot{\theta_{i}}}=Q$$

where the index *i* = 1, 2 reference the variables $\theta_{1}\left(t\right)$, $\theta_{2}\left(t\right)$, can be obtained the linearized Ordinary Differential Equations (ODEs) of motion for this two link payload free vibration given by:

(39) $$\frac{1}{24}\cdot m\cdot l^{2}\cdot \left(\left(24\cdot \alpha^{2}+8\cdot \frac{\alpha^{2}}{R}\right)\cdot \ddot{\theta_{1}}+12\cdot \alpha \cdot \ddot{\theta_{2}}\right)+m\cdot g\cdot \alpha \cdot l\cdot \left(1+\frac{1}{2R}\right)\theta_{1}=0$$

(40) $$\frac{1}{24}\cdot m\cdot l^{2}\cdot \left(8\cdot \ddot{\theta_{2}}+12\cdot \alpha \cdot \ddot{\theta_{1}}\right)+m\cdot g\cdot \frac{l}{2}\cdot \theta_{2}=0$$

where $l=l_{2}$ is the rigging length, $R=m_{2}/m_{1}$ the mass ratio and finally the additional parameter $\alpha =l_{1}/l_{2}$ depicts the lengths ratio of both links was also used. From this point the symbolic computation of Mapple 18 was employed to determine the natural frequencies depicted in Figure 11. The Figure 11 shows the two oscillation frequencies as a function of the links mass ratio *R* = $m_{2}$/$m_{1}$ and the rigging length of the second rod $l_{2}$.

According to Figure 11 keeping unchanged the mass ratio the low frequency increases as the rigging length $l_{2}$ decreases. Please note that over the range of parameters considered the low frequency varies about the 30 per cent from its mean value while for the second mode deviates about the 60 per cent.

**Figure 12:**
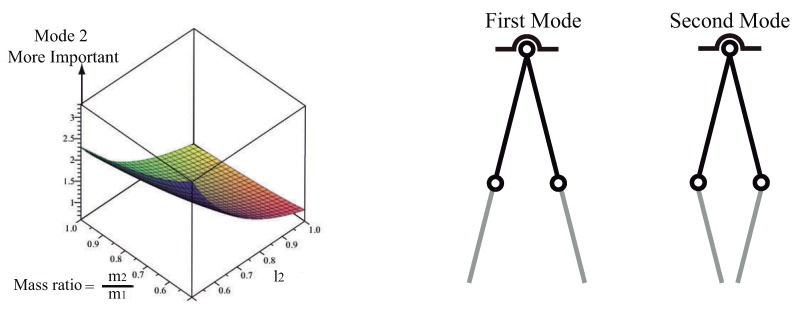
Mode Ratio High Mode to Low Mode amplitude. Conditions 0.5 < *l*${}_{2}$ < 1 and 0.5 < R < 1.

The relative contribution of both modes can be examined using the mode ratio depicted in Figure 12. The surface indicates that the second mode is most important for the multibody system dynamics when low second link to first link mass ratio occurs. The second mode contribution is particularly large when the suspension and rigging lengths are approximately equal. Recall that this is the case of the FMI where $l_{1}=0.186$ and $l_{2}=0.14$, due to the overhead crane functional mock-up interface distance limitations between the trolley and the floor. Therefore, two-mode input shaping may be necessary under these conditions.

**Figure 13:**
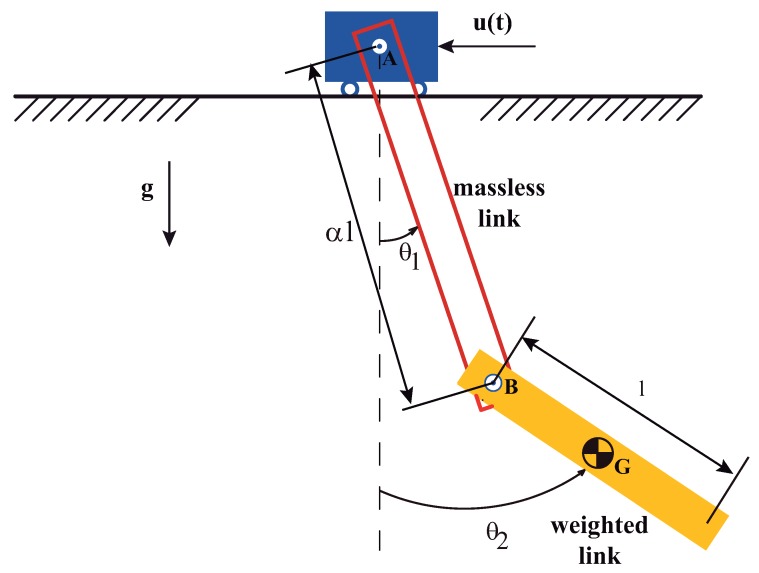
Planar double pendulum. Conditions: massless first link length α·l equivalence with composite revolute-revolute joint and the weighted second link with mass *m*, plus moment of inertia about the mass center $I_{zz}^{G}$.

Dealing with the Hydraulic crane payload let assume, because there is a wide array of cases, that the mass of the first link—usually a cable under tension—is negligible small when compared with the mass of the second link. If this is the case, the initial model may be simplified to that shown in Figure 13. This simplified model allows a much more affordable analytical approach of the system dynamics and provides fair estimates of the two natural frequencies behaviour. This estimates will be quite valuable to carry out input shapers for hydraulic cranes transporting payloads whose dynamics behaves closely to the benchmark rope plus a rod. Here the rope has been modelled as composite revolute-revolute joint shown in Figure 13.

Under these conditions from the from the Euler-Lagrange equations can be extracted the linearized ordinary differential equations (ODEs) of motion for this payload free vibration given by

(41) $$\frac{1}{24}\cdot m\cdot l^{2}\cdot \left(24\cdot \alpha^{2}\cdot \ddot{\theta_{1}}+12\cdot \alpha \cdot \ddot{\theta_{2}}\right)+m\cdot g\cdot \alpha \cdot l\cdot \theta_{1}=0$$

(42) $$\frac{1}{24}\cdot m\cdot l^{2}\cdot \left(8\cdot \ddot{\theta_{2}}+12\cdot \alpha \cdot \ddot{\theta_{1}}\right)+m\cdot g\cdot \frac{l}{2}\cdot \theta_{2}=0$$

where α is the length ratio between the massless cable and the weighted second link lengths depicted in Figure 13 and *g* is the acceleration due to gravity.

At this point, we are interested in whether the cable and link-2 can oscillate harmonically with the same frequency and phase angle but with different amplitudes. Assuming that it is possible to have harmonic motion of the cable and link-2 at the same frequency ω and the same phase angle ϕ, the solutions to Equations (41) and (42) would be

(43) $$\theta_{1}\left(t\right)=\Theta_{1}\cdot cos\left(\omega t+\phi \right)$$

(44) $$\theta_{2}\left(t\right)=\Theta_{2}\cdot cos\left(\omega t+\phi \right)$$

where $\Theta_{1}$ and $\Theta_{2}$ are the maximum amplitudes and ϕ the phase angle of $\theta_{1}\left(t\right)$ and $\theta_{2}\left(t\right)$, whose motion is not independent but is couple by the revolute joint B. Shortly, assuming a solution of the type $\theta_{i}\left(t\right)=X_{i}\cdot e^{j\omega t}$, i = 1, 2 and substituting into the two Equations (39) and (40) yields

(45) $$\frac{1}{12}\cdot \alpha^{2}l^{2}\omega^{4}-\frac{\left(3\alpha +2\right)}{6}\cdot lg\alpha \omega^{2}+g^{2}\frac{\alpha }{2}=0$$

The above equation is called the frequency or characteristic equation because solution of this equation yields two possible values for $\omega^{2}$; both real plus positive ω itself. This will be the two frequencies characteristic values of the two degree of freedom multibody pendulum system.

(46) $$\omega_{2,1}=\frac{\sqrt{\pm l\cdot \alpha \cdot (\pm 3\cdot \alpha \pm 2+\sqrt{9\cdot \alpha^{2}+6\cdot \alpha +4)}\cdot g}}{l\cdot \alpha }$$

Please note that both frequencies depend on the lengths ratio α

(47) $$\alpha =\frac{Massless-Link-Length}{Weighted-Link-Length}$$

plus the own length of the weighted second link l.

**Figure 14:**
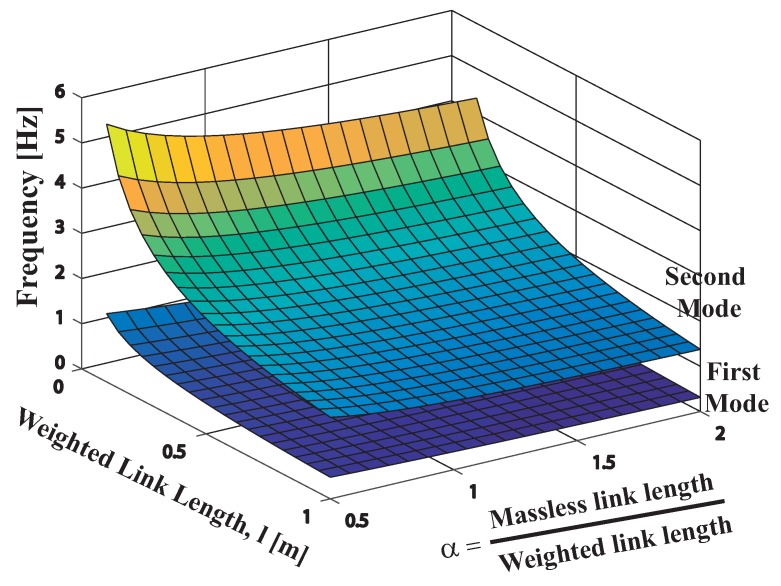
Variation of first and second mode frequencies when 0<l<1 and 0<α⩽2.

The Figure 14 shows the two oscillation frequencies as a function of (α,l) when the second link or rod length remains bellow 1 m. As shown in Table 2, the low frequency $\omega_{1}$ is maximized when both α plus *l* tend to the lowest value closed to zero. Please note that for the parameter length range [0.1–0.5 m] shown in Table 1 the standard deviation from the mean value σ is 8.84. While for the second mode, the standard deviation of the high frequency $\omega_{2}$ according to Table 3 values, reach σ = 15 over the same range for the aforementioned parameters α and *l*. In addition from Table 2 when α = 4/3 plus *l* = 0.2 $\omega_{1}$ equals 0.81 Hz this value fairly match the experimental FMI low frequency of around 1 Hz. From Table 3 when α = 4/3 keeping unchanged *l* = 0.2, $\omega_{2}$ equals 3.24 Hz somewhat higher when compared with the FMI second mode frequency of around 2.5 Hz.

These initial outcomes suggest that the control scheme for the reference input signal feed to the trolley u(t), in order to mitigate the oscillations of the modelled two link payload plus the hydarulic crane payload, would need well suited robustness dealing with the second mode. Nonetheless if the amplitudes of vibration corresponding to the second mode are exiguous when compared with the first mode then this constraint may not be critical. Anyway, to address the relative contribution of the two modes of vibration to the hydraulic crane payload swing amplitude of vibration, the mode ratio parameter

(48) $$\chi^{\left(i\right)}=\frac{\Theta_{2}^{\left(i\right)}}{\Theta_{1}^{\left(i\right)}}$$

given by the ratio of the maximum amplitudes of vibration $\Theta_{1}$, $\Theta_{2}$ might be the most appealing option.

**Figure 15:**
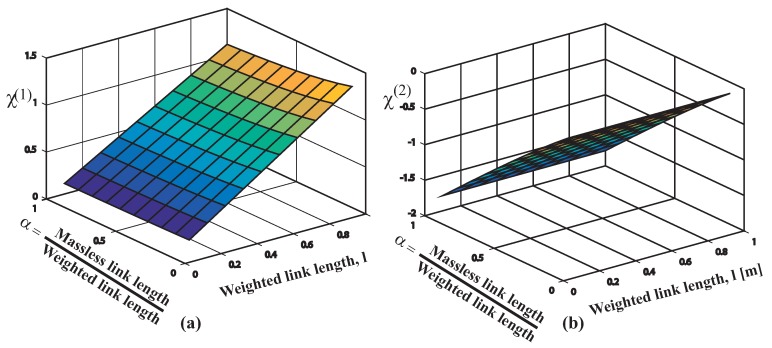
Mode ratio results: first mode $1.5\geq \chi^{\left(1\right)}>0$ both links swing in nearly in phase; second mode $0>\chi^{\left(2\right)}>-2$ both links swing in opposite phases. Conditions: l≤1 m, 0<α≤1.

The global Figure 15 shows the mode ratios $\chi^{\left(i\right)}$ *i* = 1, 2, as a function of the aforementioned range of parameters (α,l), given by

(49) $$\chi^{\left(i\right)}=\frac{\Theta_{2}^{\left(i\right)}}{\Theta_{1}^{\left(i\right)}}=\frac{\frac{1}{2}l\cdot \alpha \cdot \omega_{i}^{2}}{\frac{g}{2}-\frac{1}{3}\cdot l\cdot \omega_{i}^{2}}$$

Regarding Figure 15a the mode ratio $\chi^{\left(1\right)}$ behaves positive, if this is the case, both the cable plus the rod swing nearly in phase. Also for the range of values considered $\chi^{\left(1\right)}$ behaves less than 1.4, $\chi^{\left(1\right)}⩽1.4$ thus, in addition the amplitude of vibration $\Theta_{2}^{\left(1\right)}$ value remains closed to $\Theta_{1}^{\left(1\right)}$ value.

At this point, considering Figure 15b that depicts the mode ratio $\chi^{\left(2\right)}=\Theta_{2}^{\left(2\right)}/\Theta_{1}^{\left(2\right)}$ for the range of link-2 lengths and lengths ratio addressed, $\chi^{\left(2\right)}$ remains negative. This negative value of $\chi^{\left(2\right)}$ indicates that the cable and rod swing tendency consist in undergo opposite phases while somewhat conditioning by the initial conditions of course. In addition $0>\chi^{\left(2\right)}⩾-2$ implies that the amplitude corresponding to the second mode of vibration $\Theta_{2}^{\left(2\right)}$ become slightly greater than $\Theta_{1}^{\left(2\right)}$. The surface indicates that the mode ratio $\chi^{\left(2\right)}$ increases with high cable to rod length ratios α⩾1 keeping unchanged the condition low cable to rod mass ratios. Therefore, while somewhat limited on the scope due to the constraint applied regarding the massless cable, from these primary results the notion that a two-mode input shaping control schema would also be the most appealing option.

Finally, the system can be made to vibrate only in its ith normal mode (*i* = 1, 2) by subjecting it to the specific initial conditions. The formal normal modes correspond to $\chi^{\left(1\right)}=1$ plus $\chi^{\left(2\right)}=-1$. To attain a normal mode as response, the system might be customized solving for the parameters α plus *l*, the resulting non-linear programming is:

(50) $$\omega_{1}-\frac{\sqrt{-l\cdot \alpha \cdot (-3\cdot \alpha -2+\sqrt{9\cdot \alpha^{2}+6\cdot \alpha +4)}\cdot g}}{l\cdot \alpha }=0;$$

(51) $$\omega_{2}-\frac{\sqrt{l\cdot \alpha \cdot (3\cdot \alpha +2+\sqrt{9\cdot \alpha^{2}+6\cdot \alpha +4)}\cdot g}}{l\cdot \alpha }=0;$$

(52) $$\chi^{\left(1\right)}-1=0;$$

(53) $$\chi^{\left(2\right)}+1=0;$$

**Figure 16:**
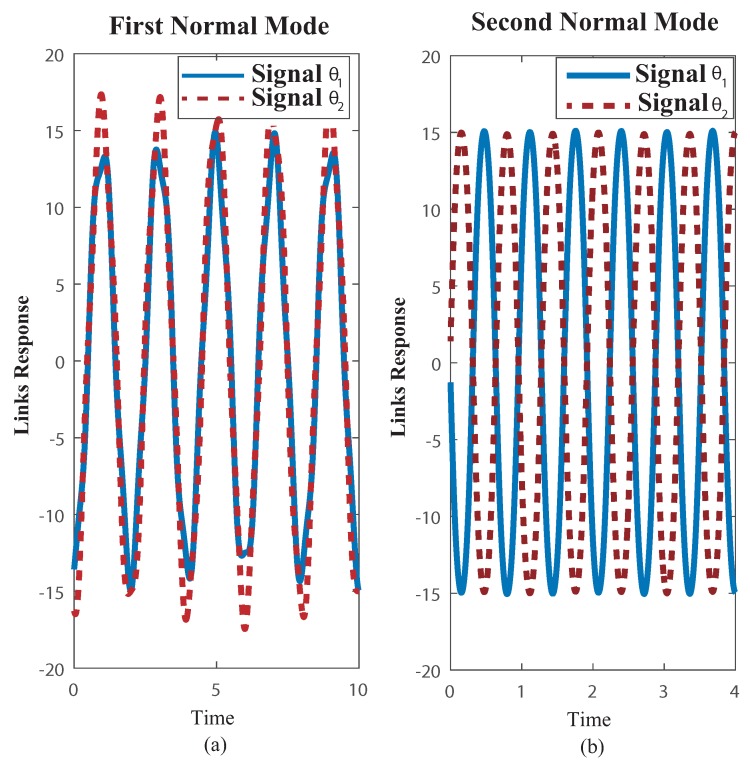
Normal Modes of Vibration. First mode both links swing closely in phase; second mode both links swing in opposite phases. Conditions: $\omega_{1}=0.5$ Hz, $\omega_{2}=1.86$ Hz, α=0.5833, l=0.8571 m.

The NLP programming converges to the following parameters values: $\omega_{1}=0.5$ Hz, $\omega_{2}=1.86$ Hz, α=0.5833, l=0.8571 m (see Figure 16). Nonetheless as it is customary, both modes will be excited for any other general conditions, the resulting motion can be obtained by a linear superposition of the two normal modes.

The next goal of this modest work addresses the Input Shaping method to define how and when the trolley of the overhead crane would move lengthwise the linear guide in order to do not induce the two link payload vibration i.e., excite any of the two modes. Dealing with the hydraulic crane what shape must have the slewing motion. If a well suited Input Shaping Filter is applied correctly, no-one of the two modes of vibration nor a linear combination of both modes described by:

(54) $$\theta_{1}\left(t\right)=\Theta_{1}^{\left(1\right)}\cdot cos\left(\omega_{1}t+\phi_{1}\right)+\chi^{\left(1\right)}\cdot \Theta_{1}^{\left(1\right)}\cdot cos\left(\omega_{2}t+\phi_{2}\right)$$

(55) $$\theta_{2}\left(t\right)=\Theta_{1}^{\left(2\right)}\cdot cos\left(\omega_{1}t+\phi_{1}\right)+\chi^{\left(2\right)}\cdot \Theta_{1}^{\left(2\right)}\cdot cos\left(\omega_{2}t+\phi_{2}\right)$$

will degrade the performances of the overhead crane or the hydraulic crane. Particularly Input Shaping performs well reducing the deviations from vertical of the multibody payload after the time of the last tap is applied plus mitigating the residual vibration at move end. This will be faced in the following.

## 6. Multimode Specified Insensitivity Input Shapers for Multibody Systems

**Figure 17:**
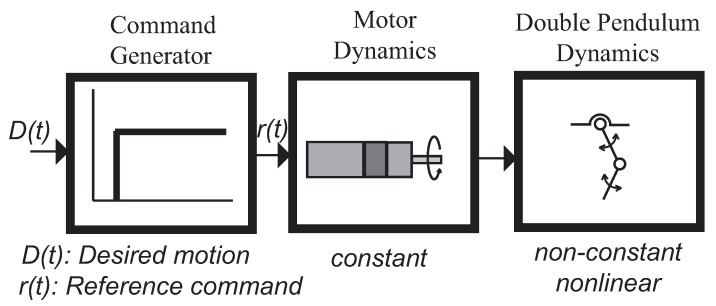
Block diagram of the generic control system.

Figure 17 shows the general block diagram of the overhead crane control system. The desired motion, *D*(*t*), is fed into a command generator usually a Joystick. The command generator transform the desired motion into a reference command *r*(*t*) which is a physical signal an analog signal dealing with the open loop FMI setup or the Hydraulic crane. Correspondingly this signal is then used to drive the servomotor or the hydraulic proportional valves. No feedback signal nor a closed-loop controller exist. However both control systems does have some form of command generator. For example, the command generator for the hydraulic crane is the human who attempts to produce an appropriate reference command in real time.

**Figure 18:**
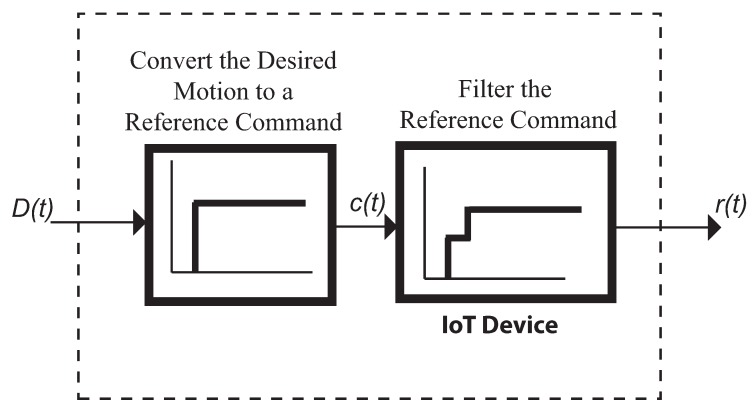
Command generator for real-time command shaping.

The preceding introduction section demonstrated the important role of the reference command. The commands that will be especially shaped for the systems under consideration will yield substantial performance improvements (residual vibration reduction) with very little cost (increase in the rise time). If this is the case the command generator can be thought of as having two distinct components as shown in Figure 18 . The first component (the Joystick) transforms the desired motion *D*(*t*) into a reference command. For example a rapid change in position is converted into a step. The second component takes into account the unwanted system dynamics and filters the reference command to produce a shaped reference command *r*(*t*). If the convolution sum to carry out the time delay filtering does not faces with a high number of filter(shaper) taps, then this process can be implemented in real time plus using an IoT device micro controller-based.

The uncomplicated way to carry out a two-mode shaper is to convolve two single mode shapers. In this case, the ZVD shapers obtained are:

(56) $$\left[\begin{array}{c} Ai\\ Ti \end{array}\right]=\left[\begin{array}{ccc} 0.25 & 0.5 & 0.25\\ 0 & 0.4545 & 0.9091 \end{array}\right]\left(1.1HzZVD\right)$$

(57) $$\left[\begin{array}{c} Ai\\ Ti \end{array}\right]=\left[\begin{array}{ccc} 0.25 & 0.5 & 0.25\\ 0 & 0.2083 & 0.4167 \end{array}\right]\left(2.4HzZVD\right)$$

Convolving the shapers given in Equations (54) and(55), generates a ZVD-ZVD given by:

(58) $$\left[\begin{array}{c} Ai\\ Ti \end{array}\right]=\left[\begin{array}{ccccccccc} 0.0625 & 0.1250 & 0.0625 & 0.1250 & 0.25 & 0.1250 & 0.0625 & 0.1250 & 0.0625\\ 0 & 0.2070 & 0.4160 & 0.4540 & 0.6610 & 0.8760 & 0.8080 & 1.1150 & 1.3240 \end{array}\right]$$

**Figure 19:**
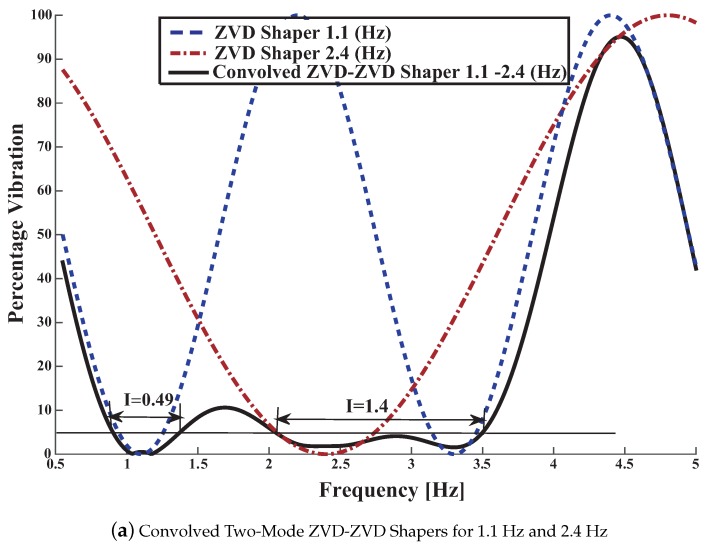

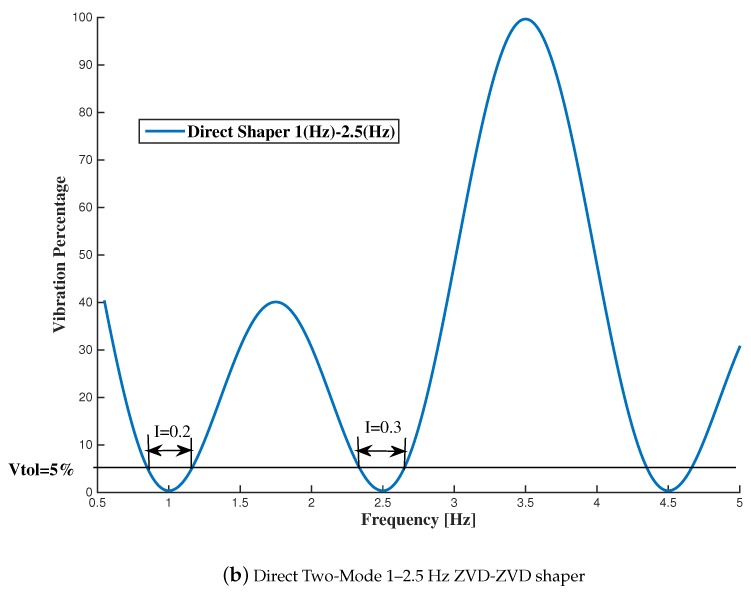
Convolved and Direct Two Mode ZVD-ZVD Shapers for 1 Hz and 2.5 Hz.

The Figure 19a corresponds to the sensitivity curves of the convolved ZVD-ZVD shaper for the range of frequencies [0.9–1.5] and [2–3.5] according to the FFT of Figure 10. The convolved shaper is very robust to modelling errors closed to the second mode 2.5 Hz frequency.

Nonetheless the price that is necessary to pay comes from the convolved shaper length: it last for 1.32 s. In addition the real time computational load in order to convolve the nine filter taps into the time credit window, that last for only 5 ms, becomes critical, because overlapping of the IoT device temporized interrupt routine may occur. Then a trade off between robustness increase and filter length must be negotiated.

Unless the convolved shapers, where the constraint equations on each mode are solved separately, the direct solution is obtained by simultaneously solving the constraint equations for both modes. To demonstrate this an example from the literature of a direct-shaper ZVD-ZVD is repeated here [35]:

(59) $$\begin{array}{c} SI_{1\mathrm{Hz}–2.5\mathrm{Hz}}=0.1673\delta \left(t\right)+0.1489\delta \left(t-0.2857\right)+0.3677 \end{array}$$

(60) $$\begin{array}{c} \delta (t-0.5714)+0.1489\delta (t-0.8571)+0.1673\delta (t-1.1429) \end{array}$$

it last for 1.143 s i.e., 0.1811 s shorter than the previous convolved ZVD-ZVD shaper. In addition it has only five taps thus the computational load is reduced. The Figure 19b shows the sensitivity curve corresponding to this Direct shaper.

Nonetheless the two-link payload can be customized in order to change the two-modes. Then let assume that the new second mode -the easiest to change- becomes closed to the 3.75 Hz. This will help to fully appreciate the direct shapers advantages, especially for mode ratios up to 3 the direct shaper properties light their own.

To this goal first a SI shaper was carried out for 1.17 Hz. A feasible solution is attained with the help of the General Algebraic Modeling System for mathematical programming and optimization (GAMS).

(61) $$SI_{1.17\mathrm{Hz}–3.51\mathrm{Hz}}=0.262500\delta \left(t\right)+0.475000\delta \left(t-0.416667\right)+0.262500\delta \left(t-0.833333\right)$$

**Figure 20:**
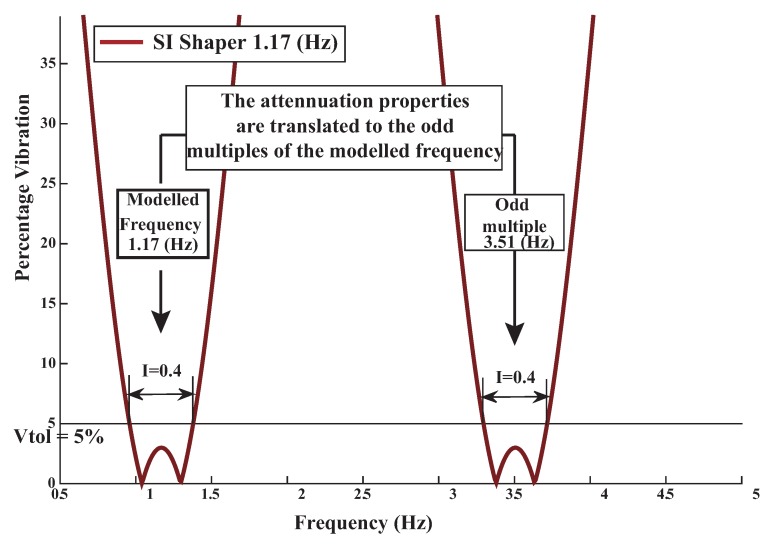
SI Shaper 1.17 Hz sensitivity curve.

The SI shaper the attenuation properties are translated to the odd multiples of the addressed frequency i.e., 3.51 Hz as demonstrated in Figure 20. Thus this shaper does not guarantees the vibration suppression at the assumed high mode frequency 3.75 Hz. A more robust solution can be attained by convolving two SI shapers for each flexible mode: 1.17 Hz plus 3.75 Hz, given by

(62) $$\begin{array}{c} CoSI_{1.17\mathrm{Hz}–3.75\mathrm{Hz}}=0.0742\delta \left(t\right)+0.121\delta \left(t-0.1690\right)+0.0681 \end{array}$$

(63) $$\begin{array}{c} \delta (t-0.3380)+0.1337\delta (t-0.4540)+0.2180\delta (t-0.6230)+ \end{array}$$

(64) $$\begin{array}{c} +0.1228\delta (t-0.7920)+0.0742\delta (t-0.9080)+ \end{array}$$

(65) $$\begin{array}{c} +0.121\delta (t-1.0770)+0.0681\delta (t-1.2460) \end{array}$$

**Figure 21:**
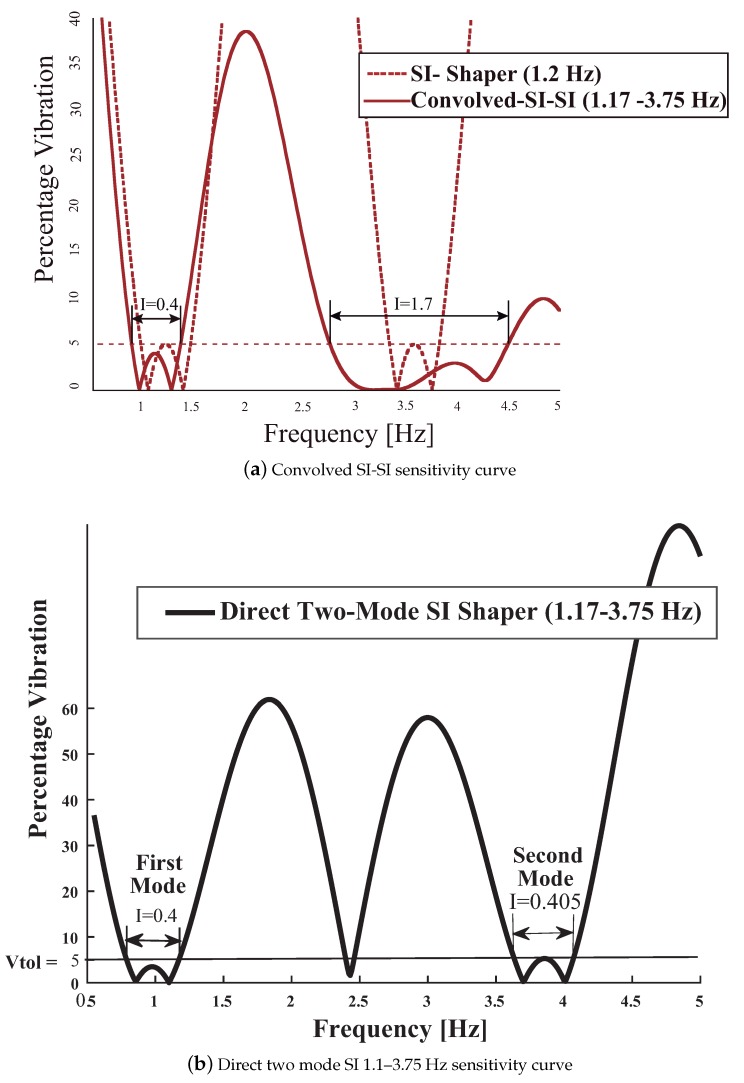
Convolved and Direct Two Mode SI-SI Shapers for 1.1 Hz and 3.75 Hz.

The sensitivity curves for the single-mode 1.17 Hz SI shaper and the Convolved (Co-SI-SI) are shown in Figure 21a. Dealing with the convolved Co-SI-SI, shaper the attenuation properties of the SI shaper for the low frequency are somewhat translated to the high frequency. However, there are some penalties: the convolved shaper contains nine impulses thus the computational requirements to perform the convolution sum at each temporized interrupt routine increase thus the time credit window of 5 ms might be insufficient. In addition the rise time delay also increases in 0.39 s. To reduce the computational requirements during run-time a Direct shaper for the two ranges of interest can be carried out. The method consist of the following stages assuming a sensitivity curve owning one hump for each mode:

- Limit the residual vibration amplitude to below Vtol at the edges or guess limits of the frequency ranges to be cancelled [(1 − I/2)$w_{mi}$ and (1 + I/2)$w_{mi}$] *i* = 1, 2.
- Set the residual vibration to Vtol at the unknown frequencies where the sensitivity curve reach one of the two local maximums.
- Set the slope of the sensitivity curve of such local maximums to zero.
- Force the residual vibration to zero at the four unknown touch frequencies $w_{zi}$ twice for each associate hump of every mode.
- Solve the aforementioned constrain equations using a proven choice for highly nonlinear problems. At this point CONOPT’s efficient and reliable multi-method architecture handles a broad range of models. Specialized techniques achieve feasibility quickly, while powerful preprocessing tools reduce problem size and suggest formulation improvements.

The Direct shaper contains only five taps,

(66) $$\begin{array}{c} DSI_{1.17Hz–3.75Hz}=0.223370\delta \left(t\right)+0.2341130\delta \left(t-0.4164916\right)+0.2843494 \end{array}$$

(67) $$\begin{array}{c} \delta (t-0.6185118)+0.2233707\delta (t-1.0339999)+0.0347959\delta (t-1.2360201) \end{array}$$

**Figure 22:**
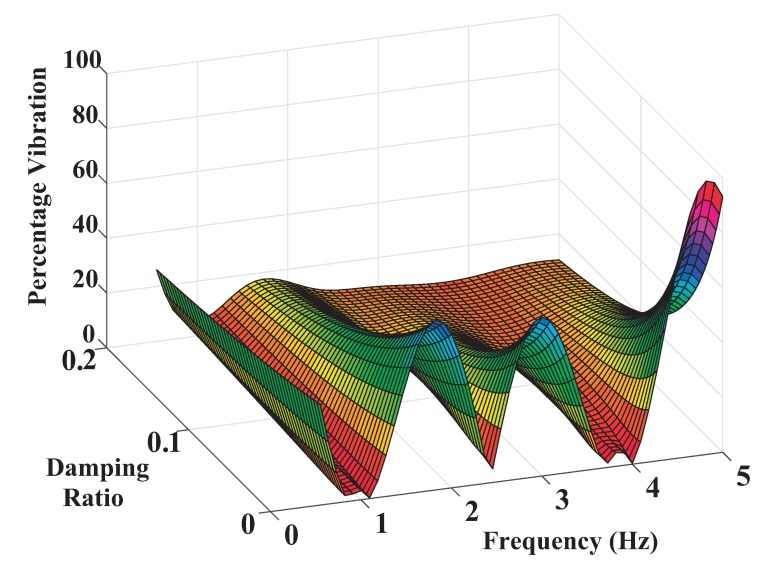
Three-Dimensional Sensitivity Curve of SI-SI Shaper Designed with Vtol = 5 percent, I = 0.5 and Suppressed Frequency Ranges 1.1–3.75 Hz.

The computational requirements are reduced when compared with the convolved shaper. In addition this shaper keeps unchanged the robustness I = 0.4 at the shifted high mode 3.75 Hz. The Figure 21b depicts the sensitivity curve corresponding to the Direct Shaper. As expected, this shaper mitigates the amplitude of the residual vibration over the two ranges of interest (see Figure 22).

## 7. Embedded Input Shaping Design Methodology and Experimental Results

The Embedded Input Shaping Design consist in set-up a three state petri-network on an mbed IoT device. The first state discriminates an unwanted action over the Joystick from a planned motion action. The minimum time that the operator activate the Joystick should be greater than the Direct Shaper length in order to consider a planned motion action. The second state initiates the shaping process using the stored input values into a memory pool those values are convolved with the Direct SI shaper taps. Finally once the operator deactivates the Joytstick the convolution sum still runs until the time delay induced by the filter length is completed, thus the last petri-network third state is finished and the finite state machine or petri network come back to state one.

**Figure 23:**
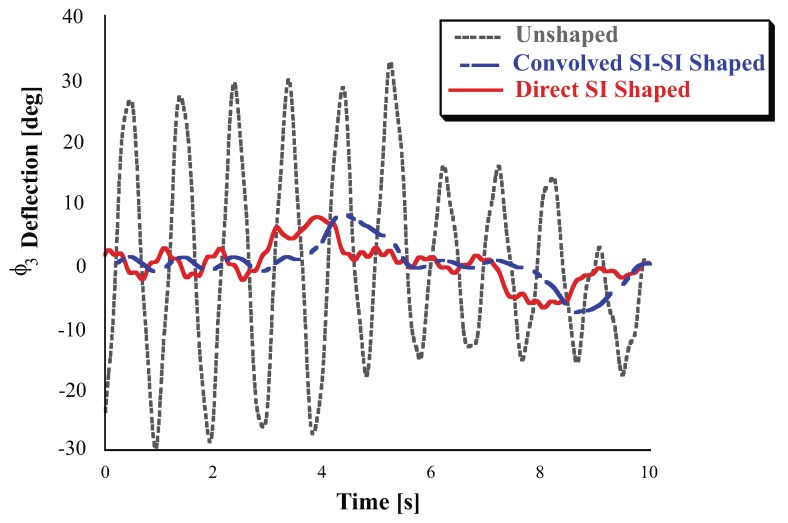
Comparison of Direct SI Shaped and Unshaped $\phi_{3}$ or $\theta_{2}$ Deflection-Limited and Unlimited Responses.

The Experimental results shown here will be presented by modulating the baseline trapezoidal velocity profile command signal of the trolley, which has the following parameters: unshaped slope $\ddot{x_{1}}$ = 0.8 m/s${}^{2}$, final velocity $1\frac{m}{s}$, rise time 1.25 s. The Convolved SI-SI shaper duration is 1.2460 s. The overall Figure 23 shows the responses when the trolley of the FMI undergoes the unshaped baseline velocity profile the Direct and the Convolved SI-SI Shaped velocity profiles. The second-link deflection has been chosen as the most descriptive variable to examine the video tapping responses. These responses are labeled with Unshaped, Direct Shaped and Convolved SI-SI Shaped marked with discontinuous line for the first case, continuous line for the second case and a hypen-line for the third case. Significant vibration exists for the unshaped response, the $\phi_{3}$ (generalzed coordinates) or $\theta_{2}$ (generalized coordinates) angle have a wide variation. For the Direct-shaped case the $\phi_{3}$ keeps close to its rest position. Please note that for the Convolved SI-SI case, the response is delayed in time regarding the Direct SI shaper. Nonetheless, as expected for the same range of kinematic parameters the shaped residual vibration always remains small. If the shaped vibration amplitude is divided by the unshaped vibration, then the resulting percentage of vibration is less than the 5%.

The experiments were conducted on the mini-overhead crane FMI called MUTOC (Multibody Two Link payload Overhead Crane). MUTOC consists of a rack fixed to the frame of reference, a linear bearing guide 1.5 m long whose trolley is actuated by an Accurax G5 Omron servomotor that is fastened firmly to the trolley by a bracket plus a miniature T-slot structure licensed by Markerbeam. To the servomotor shaft a gear was fixed using a parallel key.

**Figure 24:**
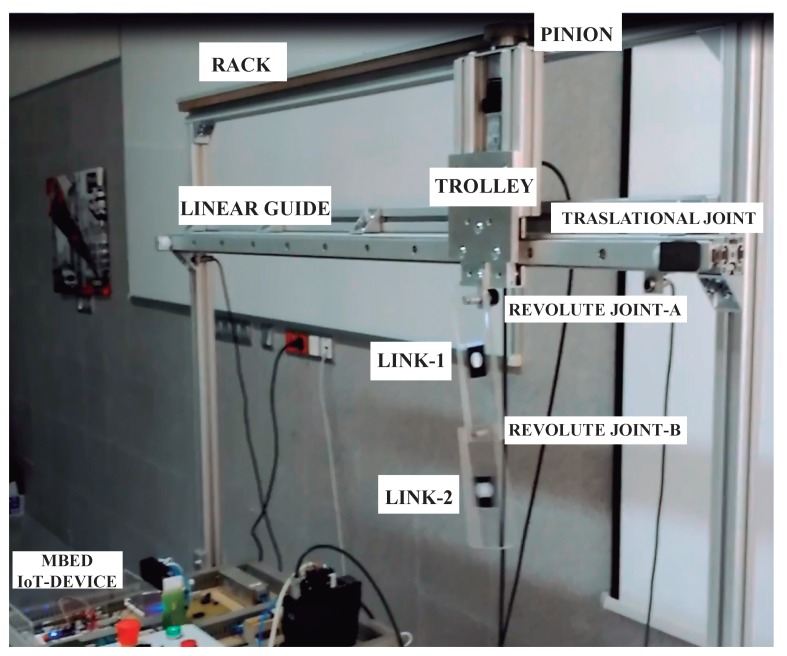
Mini Overhead Crane functional mock-up interface.

A revolute rolling element bearing joint is based between the trolley and the first link, without any rotary sensor do not measures the deflection from the vertical of the first link, i.e., the angle $\phi_{2}$, in order to do not introduce coulombian friction or any additional inertial parameter. Instead, a web camera video tape recording at 60 frames per second the whole motion, will allow the estimation of the angle $\phi_{2}$ plus the second link body deflection angle $\phi_{3}$ relative to frame of reference. By extracting the mass centres of the bodies plus the joints positions at each video frame, the corresponding deflections of both links are carried out by a Matlab script that uses the Image Processing Toolbox (see Figure 24).

Thus, the Omron RD-88 servomotor-driver is connected to the mbed microcontroller via D/A converter . The velocity baseline signal is generated trough C code run by the mbed. MUTOC is able to move attending the analog JoyStick commands feed to the mbed. Direct Input Shaper data was introduced into the mbed C-program in order to carry out in real time the convolution sum. To this goal a temporized interrupt subroutine with five milliseconds time credit window was programmed as in [36].

**Figure 25:**
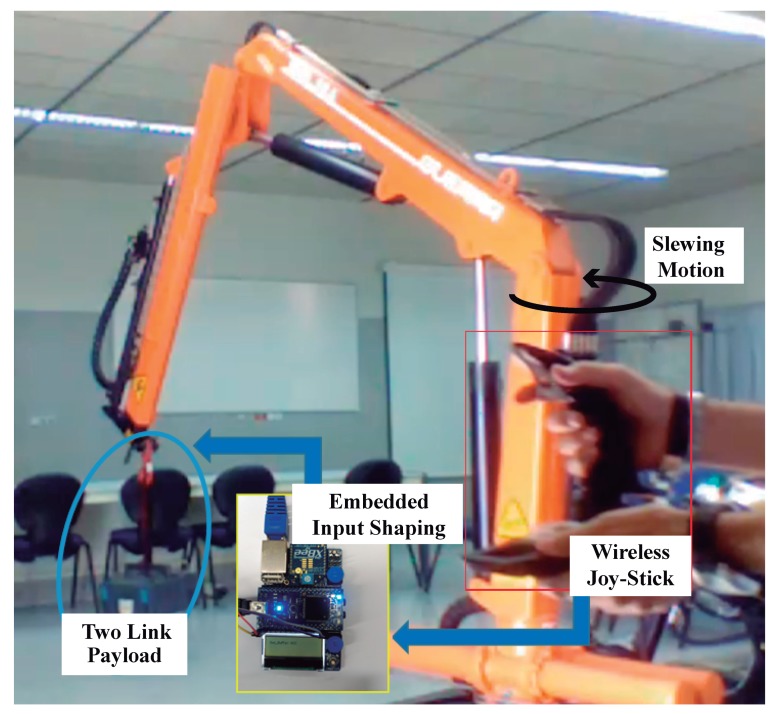
The hydraulic crane at the laboratory for the test.

**Figure 26:**
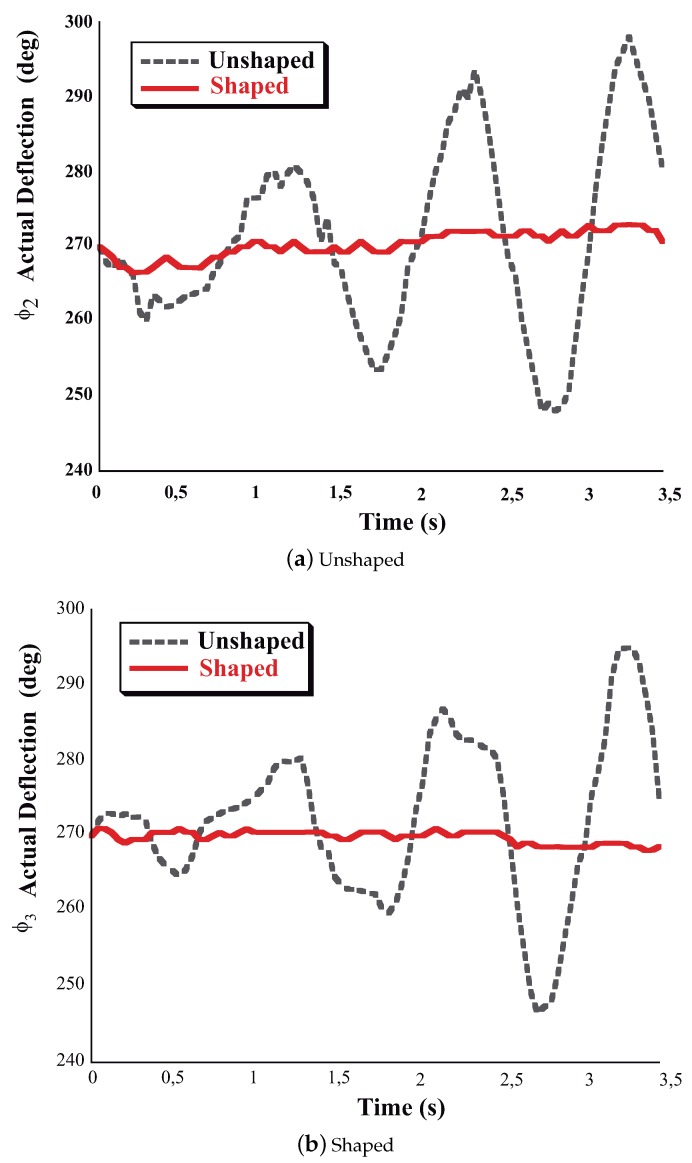
Unshaped and Direct SI-Shaped Experimental Responses of the Hydraulic crane under slewing motion. Conditions: sampling frequency 60 Hz.

At this point the company Guerra Industries grant our laboratory an hydraulic crane shown in Figure 25 well suited to interface with it the aforementioned hardware adding a pair of XBee-ZigBee radio modules the crane was moved performing the slewing motion, including ramp up and ramp down phases, for more than 3.5 s through its workspace. The responses to the unfiltered and filtered commands can be seen in the overall Figure 26. The unfiltered command, Figure 26a, causes significant deviations of the links from vertical. However, as it can be seen the Direct SI-Filtered command mitigates the vibration of both links depicted in Figure 26b. Regarding the percentage of residual vibration to be honest only a small amount of vibration of around the 10 percent dealing with the angle $\phi_{\left(2\right)}$ plus a 7 percent dealing with the angle $\phi_{\left(3\right)}$ still remains due to some actuator dead-zones plus hydraulic system uncertainties [37].

## 8. Conclusions

For the wide array of overhead cranes composed by bodies plus kinematic joints, good candidates to implement Input Shaping, a multibody model would be the most appealing option better than a particle oriented model. A method oriented to the application of Input Shaping dealing with actual multibody systems whose dynamics is better described by DAEs has been presented. For these systems the identification of the natural frequencies is not as straight forward to obtain as in the case of linear systems described by ODEs, coming from simple particle oriented models sometimes unaccurately.

The reliability of the multibody model was tested on a functional mock up interface (FMI) with a two link payload suspended from the trolley. The FFTs of the simulated and the experimental signal contain the same frequency harmonics only with somewhat different power due to the real world light damping in the joints. To give some insight into the multibody double pendulum dynamics by Lagrangian methods a two link benchmark multibody model is proposed. This allows to develop a general study of this type of multibody payloads dynamics including its normal modes, modes ratios plus ranges of frequencies expected. Also for wide array of hydraulic cranes if the payload size is not negligible small when compared with the cable length may introduce an additional oscillatory mode that creates a multibody double pendulum. A somewhat simplified multibody model is proposed assuming a composite revolute-revolute joint to model the overhead cable.

Input Shaping has been adopted as feedforward strategy to do not induce unwanted multibody payload dynamics by shaping the reference input feed to trolley motion lengthwise the linear guide. To this goal, Convolved and Direct Specified Insensitive shapers that attenuate the vibration at the necessary ranges of frequency have been carried out. The log of the experimental real time deflection for the unshaped and shaped responses demonstrates clearly the beneficial effect of applying input shaping in the command generator. The command generator consist on an Internet Of Things device 32 bits microcontroller based. The Embedded Input Shaping Design consist in set-up a three state petri-network on the mbed IoT device. The first state discriminates an unwanted action over the Joystick from a planned motion action. The minimum time that the operator activate the Joystick should be greater than the Direct Shaper length in order to consider a planned motion action. The second state initiates the shaping process using the stored input values into a memory pool those values are convolved with the Direct SI shaper taps. Finally once the operator deactivates the Joytstick the convolution sum still runs until the time delay induced by the filter length is completed, thus the last petri-network third state is finished and the finite state machine or petri network come back to state one. This IoT command generator was tested on the overhead crane FMI and the hydraulic real-world crane hanging distributed multibody playloads.

## Figures and Tables

**Table 1:** Simulation Parameters.

Multibody Model Parameter	Value
$\xi_{2}^{A}$	−0.0825 (m)
$\xi_{2}^{B}$	0.0825 (m)
$\xi_{3}^{B}$	−0.070 (m)
$I_{zz}^{2}$	2.26875 × $10^{-4}$ (Kgr·m${}^{2}$)
$I_{zz}^{3}$	1.21422 × $10^{-4}$ (Kgr·m${}^{2}$)
$m_{1}$	585 (gr)
$m_{2}$	99.12 (gr)
$m_{3}$	74.34 (gr)

**Table 2:** First Mode frequency $\omega_{1}$ (Hz).

	$\alpha =\frac{1}{2}$	α=1	α=4/3	α=2
Length [m]	•	•	•	•
*l* = 0.1	1.5097	1.2622	1.1491	0.9900
*l* = 0.2	1.0675	0.8925	0.8125	0.7001
*l* = 0.3	0.8716	0.7287	0.6634	0.5716
*l* = 0.4	0.7548	0.6311	0.5745	0.4950
*l* = 0.5	0.6751	0.5645	0.5139	0.4428

**Table 3:** Second mode frequency $\omega_{2}$ (Hz).

•	α	α=1	α=4/3	α=2
Length [m]	•	•	•	•
*l* = 0.1	•	4.8224	4.5873	4.3473
*l* = 0.2	•	3.4100	3.2437	3.0740
*l* = 0.3	•	2.7842	2.6485	2.5099
*l* = 0.4	•	2.4112	2.2937	2.1737
*l* = 0.5	•	2.1567	2.0515	1.9442

## Abbreviations

The following abbreviations are used in this work:

*ODEs*	ordinary differential equations
*DAEs*	differential algebraic equations
$s_{i}^{p}$	the vector since the origin of the local frames to the point
*ŭ*	the vector u rotated counterclockwise 90°
$\xi_{i}$	local *x*-axis of body *i* at the mass centre
$\eta_{i}$	local *y*-axis of body *i* at the mass centre
$\phi_{i}$	rotational coordinate of body *i*
$x_{i}$	*X* component of the mass centre of body *i*
$y_{i}$	*Y* component of the mass centre of body *i*
Φ(c)	joints constraints equations
$\dot{\Phi }$	velocity constraints equations
*D*	Jacobian of the velocity constraints equations
γ	the (right-hand side) of the acceleration constraints
*M*	array of inertial properties of the bodies
*h*	external forces
λ	Lagrange multipliers
$\theta_{i}$	independent coordinate *i*
*A*	State Space Jacobian matrix
ZV	Zero Vibration Shaper
ZVD	Zero Vibration and Derivative Shaper
SI	Specified Insensitivity Shaper
FMI	Functional Mock-Up Interface
